# Supplementary material for: Auditing the fairness of the US COVID-19 forecast hub’s case prediction models
Source: PLoS One. 2025 Apr 22;20(4):e0319383. doi: 10.1371/journal.pone.0319383 (PMC12014149; doi:10.1371/journal.pone.0319383)
Supplement: S1 Appendix — (PDF) [file pone.0319383.s001.pdf]

S1 Appendix: Supporting Information for *Auditing the Fairness of COVID-19 Forecast Hub's Case Prediction Models*

Saad Mohammad Abrar,
Naman Awasthi
Daniel Smolyak,
Vanessa Frias-Martinez

This appendix contains supplementary information that is not an essential part of the text itself but which may be helpful in providing a more comprehensive understanding of the research problem or it is information that is too cumbersome to be included in the body of the paper.

1 Supporting Information for Data

1.1 Exclusion of some counties and certain minority races

Our analysis excluded certain counties and racial/ethnic groups based on data availability and statistical considerations. At the county level, we excluded three geographical regions:

- Florida counties were excluded due to insufficient data in the PLACES Dataset, which did not meet the CDC's minimum data requirements for inclusion[1].
- Connecticut counties were excluded due to a structural mismatch in reporting systems. Connecticut's switch from county FIPS reporting to planning region reporting created incompatibilities between COVID-19 ForecastHub predictions, PLACES dataset, and ACS demographic data[2].
- Puerto Rico counties (FIPS Code: 72XXX) were excluded as the COVID-19 ForecastHub does not provide county-level forecasts for this territory.

Regarding racial and ethnic groups, we made methodological decisions based on population distributions across U.S. counties (see Table 1 in main manuscript). We excluded American Indian/Alaska Native (AIAN) and Native Hawaiian/Pacific Islander (NHPI) populations from our regression analysis due to limited variable distribution. Specifically, 97.69% of counties have NHPI populations under 1%, and 79.03% have AIAN populations under 1%, providing insufficient variation for meaningful regression analysis.

In contrast, over one-third of Asian counties, two-thirds of Black counties and almost all Hispanic counties have more than 1% of population presence. Additionally, while Asian populations show a relatively low median of 0.70% (compared to Black and Hispanic counties), their mean percentage (1.53%) is more than twice the median, indicating a right-skewed but analytically useful distribution. These statistics reveal an adequate statistical power for detecting potential effects in our regression analysis. Furthermore, Asian populations represent a key demographic group in metropolitan and suburban areas where COVID-19 impacts were particularly pronounced, making their inclusion crucial for understanding prediction accuracy in these important contexts.

1.2 Description of the model type classification

The authors of this paper created a model taxonomy (see below) after reading the papers for each of the 36 models under study. Using this taxonomy, each COVID-19 forecast model was labeled by two authors of this paper. Whenever there was a labeling disagreement, the model was discussed until a final agreement was reached. For transparency purposes, we provide the complete classification of all models along with their descriptions in Table 1 in this Appendix.

We propose the following refined model taxonomy based on the primary modeling approach used by each team:

- **Compartmental Models:** Models that primarily use variations of epidemiological compartmental frameworks (e.g., SIR, SEIR) as their core predictive mechanism. While these models may incorporate additional

[1] `https://www.cdc.gov/places/current-release-notes/index.html#:~:text=2023%20Release,were%20carried%20over%20for%20Florida`

[2] `https://developer.ap.org/ap-elections-api/docs/CT_FIPS_Codes_forPlanningRegions.htm`

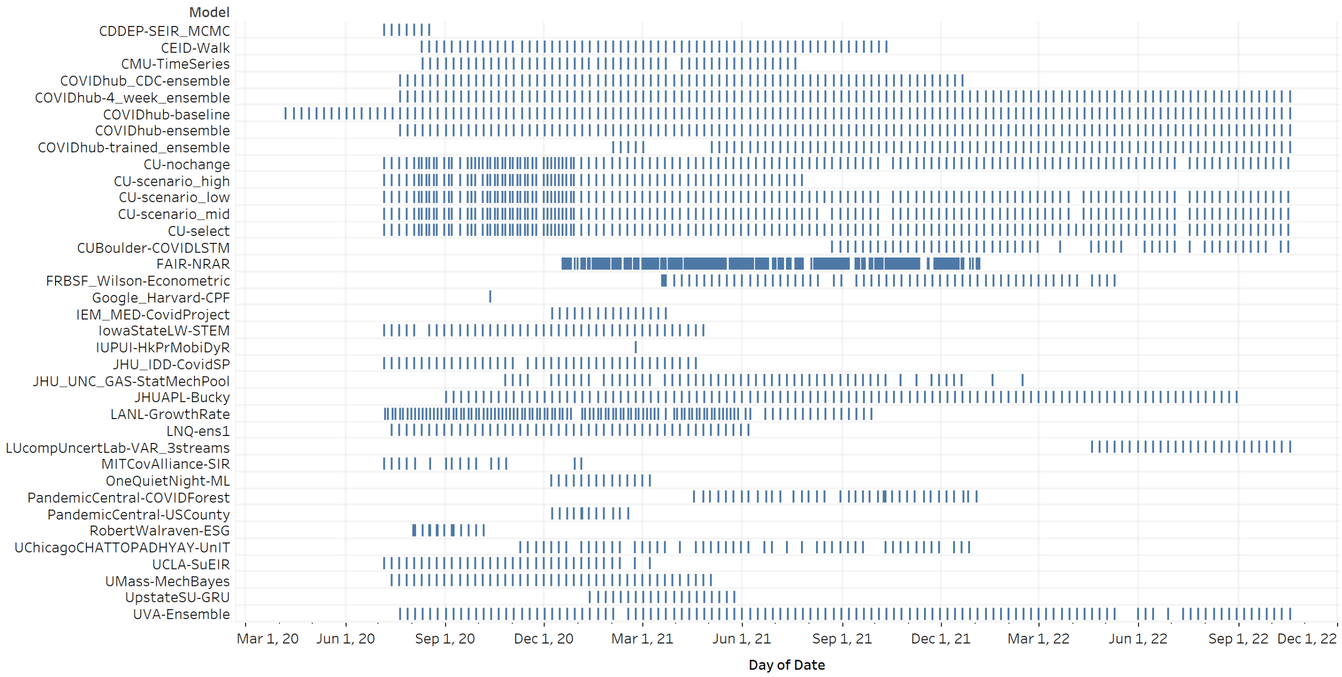

Figure 1: **Timeline Overview of COVID-19 Model Forecasts by County.** This Gantt chart captures the forecast submission frequency from the 36 research teams' models analyzed in this paper, delineating the evolution of modeling efforts throughout the pandemic.

computational techniques for parameter estimation, they are classified based on their fundamental epidemiological structure. For example, UCLA-SuEIR uses BFGS optimization with an SEIR model, but its primary predictive mechanism remains compartmental. Similarly, while the IEM_MED-CovidProject model uses AI techniques for parameter fitting, we classify it as compartmental because its core predictive mechanism relies on a SEIR framework, with AI serving an auxiliary role in parameter optimization. This classification prioritizes the fundamental epidemiological structure over supplementary computational techniques used for implementation.

- **Statistical Models:** Models that rely predominantly on statistical methods including time series analysis, regression, and classical statistical approaches. For instance, CMU-TimeSeries uses quantile regression, while CEID-Walk employs random walk methods.

- **Deep Learning Models:** Models that primarily leverage neural network architectures for prediction. For example, CUBoulder-COVIDLSTM uses LSTM networks as its core prediction mechanism, while Google_Harvard-CPF employs deep learning with mobility data.

- **Ensemble Models:** Models that systematically combine predictions from multiple forecasting approaches. We identify two types of ensembles in the Forecast Hub: (1) Hub-level ensembles (e.g., COVIDhub_CDC_ensemble, COVIDhub-ensemble, etc) that aggregate predictions across different participating teams' models, and (2) team-level ensembles (e.g., UVA-Ensemble, LNQ-ens1) that combine multiple methodological approaches within a single submission. For example UVA-Ensemble computes a bayesian model averaging of autoregressive models with mobility data, LSTM networks, and modified SEIR models. The common thread across both types is their systematic integration of multiple predictive approaches to generate final forecasts.

- **Baseline Models:** Models that serve as fundamental comparison benchmarks. In the Forecast Hub, the primary baseline model (COVIDhub-baseline) uses a specific procedure where the median prediction equals the most recent observed incidence, with uncertainty derived from historical week-to-week changes. This approach assumes future incidence will be similar to current levels, with variations based on historically observed changes. This provides a simple but statistically grounded reference point against which to compare more sophisticated approaches, and is classified as a Baseline model in our approach.

This taxonomy acknowledges that modern epidemiological models often incorporate multiple computational techniques, but classifies them based on their primary predictive mechanism rather than auxiliary methods used for implementation or optimization. We recognize that some models may exhibit hybrid characteristics, but we assign them to categories based on their dominant modeling paradigm to enable meaningful comparative analysis.

Model Name	Model Type	Mobility Used?
CU-nochange	Compartmental	Yes (Census)
CMU-TimeSeries	Statistical	No
COVIDhub_CDC-ensemble	Ensemble	Mixed
COVIDhub-trained_ensemble	Ensemble	Mixed
COVIDhub-ensemble	Ensemble	Mixed
IEM_MED-CovidProject	Compartmental	No
LNQ-ens1	Ensemble	No
JHU_IDD-CovidSP	Compartmental	No
CUBoulder-COVIDLSTM	Deep Learning	Yes (Facebook)
Google_Harvard-CPF	Deep Learning	Yes (Descartes Labs)
FRBSF_Wilson-Econometric	Compartmental	Yes (Google and Safegraph)
UMass-MechBayes	Statistical	No
LANL-GrowthRate	Compartmental	No
UCLA-SuEIR	Compartmental	No
COVIDhub-4_week_ensemble	Ensemble	Mixed
CEID-Walk	Statistical	No
RobertWalraven-ESG	Statistical	No
OneQuietNight-ML	Statistical	Yes(Apple, Google)
CU-scenario_high	Compartmental	Yes (Census)
IUPUI-HkPrMobiDyR	Statistical	Yes (Google)
CDDEP-SEIR_MCMC	Compartmental	No
UpstateSU-GRU	Deep Learning	Yes (Google)
CU-scenario_mid	Compartmental	Yes (Census)
CU-scenario_low	Compartmental	Yes (Census)
UChicagoCHATTOPADHYAY-UnIT	Statistical	No
PandemicCentral-USCounty	Statistical	Yes (Facebook)
JHUAPL-Bucky	Compartmental	Yes (Safegraph)
COVIDhub-baseline	Baseline Models	Not Applicable
PandemicCentral-COVIDForest	Statistical	Yes (Facebook)
LUcompUncertLab-VAR_3streams	Statistical	No
FAIR-NRAR	Deep Learning	Yes (Facebook, Google)
JHU_UNC_GAS-StatMechPool	Ensemble	Yes
CU-select	Compartmental	Yes (Census)
IowaStateLW-STEM	Compartmental	Yes (Descartes Labs, US DOT)
MITCovAlliance-SIR	Compartmental	Yes (Safegraph)
UVA-Ensemble	Ensemble	Yes

Table 1: List of the **36 COVID-19 forecast models** analyzed in this paper, together with their model type and whether they used mobility data or not, including the source of that mobility data. Information about model type and the use of mobility data was manually extracted from the paper(s) for each model by the authors of this paper.

1.3 Description of Phases

We divided the pandemic timeline into distinct phases P using a data-driven approach based on local minima (valleys) and local maxima (peaks) in COVID-19 case volumes. This segmentation was motivated by several methodological and epidemiological considerations:

- **P0 (June 3, 2020 - September 9, 2020):** Captures the initial study period with relatively stable low case numbers. The phase ends at a clear valley (around 40,000 cases) before the first major wave, providing a baseline period for model performance analysis.
- **P1 (September 9, 2020 - March 7, 2021):** Encompasses the first major wave reaching approximately 250,000 weekly cases. The start and end points are defined by distinct valleys, capturing a complete epidemic cycle from rise through peak to decline, allowing for the analysis of model behavior during sustained transmission increase.
- **P2 (March 7, 2021 - June 23, 2021):** Represents an inter-wave period with moderate case levels. Both boundaries are marked by clear valleys, isolating a period of relative stability between major waves, which enables assessment of model performance during more stable epidemic conditions.

- **P3 (June 23, 2021 - November 3, 2021):** Contains a distinct wave reaching around 170,000 cases. The phase boundaries capture the full progression from valley through peak to subsequent valley, encompassing the Delta variant wave period.
- **P4 (November 3, 2021 - March 30, 2022):** Defined to contain the most dramatic surge in the dataset, reaching approximately 800,000 cases. This phase captures the Omicron wave from its onset through peak to initial decline, allowing analysis of model performance during extreme case volumes.
- **P5 (March 30, 2022 - June 22, 2022):** Represents the sharp post-Omicron decline phase. The boundaries isolate this distinct period of rapid case reduction, enabling analysis of model behavior during sustained decrease.
- **P6 (June 22, 2022 - October 20, 2022):** Final phase containing a smaller but distinct wave (Around 130,000 cases). The boundaries capture this complete wave cycle, allowing assessment of model performance during later pandemic dynamics.

This valley-based segmentation offers several analytical advantages. Each phase represents a complete epidemiological pattern (rise-peak-fall or relative stability), with phase transitions occurring at natural breakpoints in case dynamics. The segmentation captures varying intensities of transmission, from baseline to extreme peaks, and phases align with known variant waves while remaining data-driven rather than variant-defined. Additionally, the approach provides sufficient samples within each phase for robust statistical analysis. This systematic segmentation enables us to evaluate how model fairness metrics vary not only across different case volumes but also across distinct epidemic patterns and trajectories throughout the pandemic period.

2 Supporting Information for Preprocessing & Model selection

2.1 Data Preprocessing

Initial exploratory analysis of the Pinball Loss norm (PBL) distribution revealed several characteristics that influenced our modeling choices. The original distribution exhibited extreme right-skewness with a heavy tail, as evidenced by the substantial difference between the bulk of the distribution and its maximum value (see Figure 2, top left) and Table 2. This pattern is typical of error metrics but poses challenges for standard linear regression assumptions.

To address these distributional characteristics, we explored two key preprocessing steps:

1. **Data Trimming:** We observed that the top 1% (14,672 out of 1,541,541 observations) of PBL values were substantially higher than the rest of the distribution (difference between the 99th percentile (1.47×10^{-3}) and the maximum value (5.18×10^{-1})), potentially exerting excessive leverage in regression models. Trimming these extreme values while preserving 99% of observations (Figure 2, bottom left) improved the stability of the distribution without sacrificing the majority of the data points.
2. **Square Root Transformation:** To further address the remaining right-skewness, we applied a square root transformation to the PBL values. This transformation significantly improved the distribution's properties, producing a more symmetric shape that better satisfies regression assumptions (Figure 2, bottom right).

**Quantile**	**Pinball Loss Norm**
50th (Median)	1.96×10^{-4}
75th	3.41×10^{-4}
90th	5.53×10^{-4}
95th	7.46×10^{-4}
97th	9.22×10^{-4}
98th	1.09×10^{-3}
99th	1.47×10^{-3}
Maximum	5.18×10^{-1}

Table 2: Distribution of Pinball Loss Norm Values

Given these distributional characteristics and the strictly positive nature of our dependent variable, we tested both Gamma and Gaussian GLM families with log-link and identity-link functions. Alternative model specifications, including Gaussian GLMs with identity link and Gamma GLMs with log and inverse link, were evaluated but provided inferior fit based on diagnostic plots, residual patterns, and model performance metrics. The final choice of Gaussian GLM with log link function applied to square root transformed data (with 1% trimming) best addressed both the

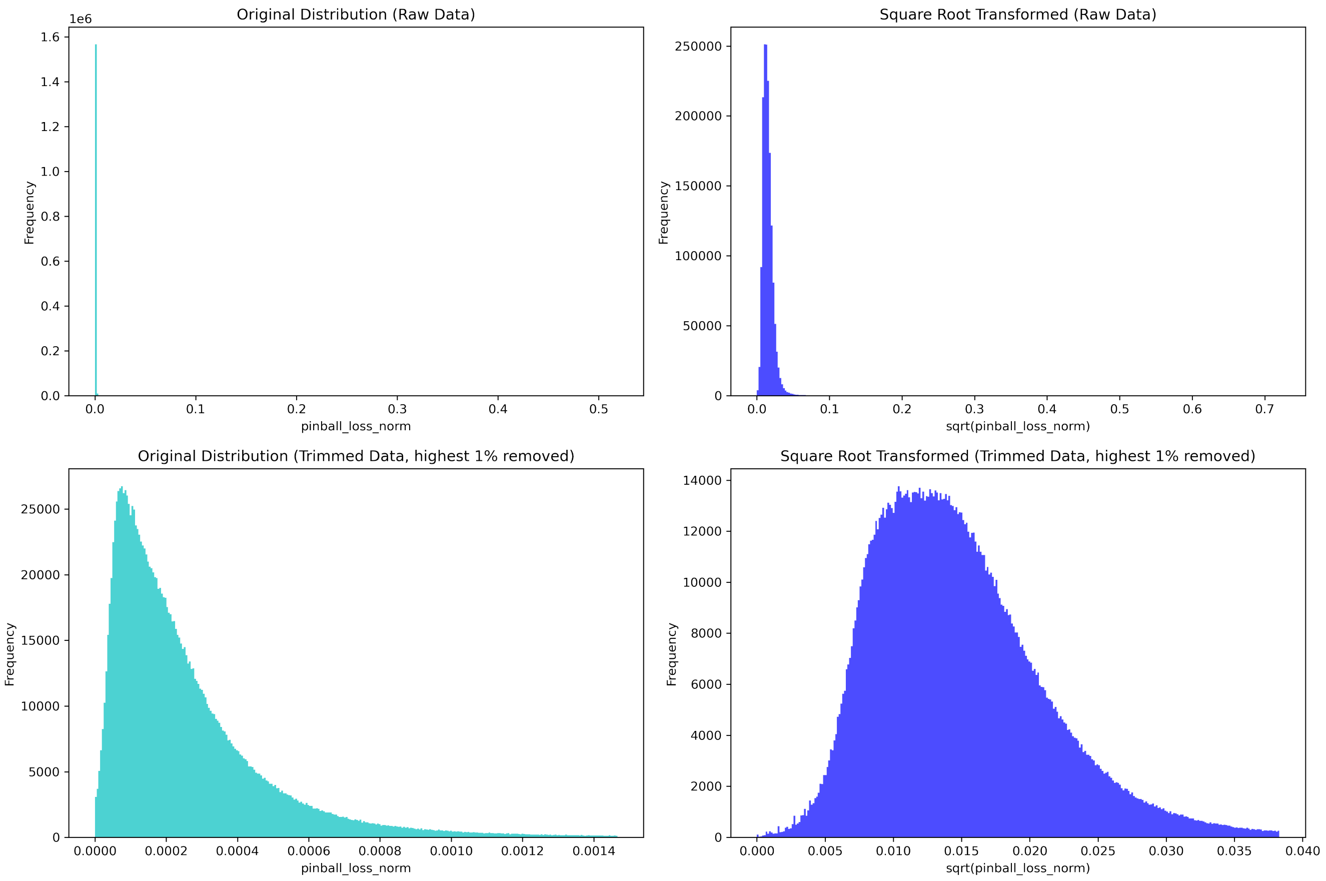

Figure 2: **Distribution of the PBL metric** used to evaluate COVID-19 case prediction errors. Top row shows the original (left) and square root transformed (right) distributions of the raw PBL data. Bottom row shows the same distributions after trimming the highest 1% of values, demonstrating improved visualization of the data's shape and reduction of extreme outliers.

distributional characteristics of our dependent variable and the heteroscedasticity observed in initial diagnostics. Specific model diagnostics are discussed in Section 2.3.

After evaluating diagnostic plots, residual patterns, and model performance metrics (pseudo-R^2 values), the Gaussian GLM with log-link function applied to the square root transformed data (with 1% trimming) provided the best fit. This combination effectively addressed both the heavy-tailed nature of the original distribution and the heteroscedasticity observed in initial model diagnostics.

	Iteration 1		Iteration 2		Iteration 3		Iteration 4		Iteration 5		Iteration 6		Iteration 7	
Variable	Raw_GVIF	A_GVIF	Raw_GVIF	A_GVIF	Raw_GVIF	A_GVIF	Raw_GVIF	A_GVIF	Raw_GVIF	A_GVIF	Raw_GVIF	A_GVIF	Raw_GVIF	A_GVIF
black_perc	11.36818	3.371673	9.767423	3.125288	9.296005	3.048935	6.806963	2.609016	4.407688	2.099449	2.592352	1.610078	1.321822	1.149705
hispanic_perc	8.925986	2.987639	7.617554	2.759992	6.75456	2.598954	6.385277	2.526911	6.37993	2.525852	2.474057	1.572914	1.159437	1.076772
asian_perc	2.06554	1.437199	2.065375	1.437141	1.918433	1.385075	1.918099	1.384955	1.917993	1.384916	1.457816	1.2074	1.456597	1.206896
factor(lookahead)	1.002012	1.000335	1.002011	1.000335	1.002011	1.000335	1.00201	1.000335	1.00201	1.000335	1.00201	1.000335	1.002006	1.000334
factor(phase)	1.113057	1.008966	1.113048	1.008965	1.113046	1.008965	1.113028	1.008964	1.113026	1.008963	1.113015	1.008963	1.112995	1.008961
factor(model_type)	3.461884	1.167923	3.461752	1.167917	3.461675	1.167914	3.461639	1.167912	3.461401	1.167902	3.461395	1.167902	3.461362	1.167901
factor(mobility_used)	3.529834	1.370688	3.529651	1.37067	3.529602	1.370665	3.529574	1.370663	3.529503	1.370656	3.529393	1.370645	3.529174	1.370624
~~BPHIGH~~	12.871	3.587617	12.65528	3.557427	10.84353	3.292951	9.612044	3.10033	NA	NA	NA	NA	NA	NA
~~CANCER~~	9.636473	3.104267	9.61278	3.100448	9.537255	3.088245	9.537094	3.088219	9.53316	3.087582	NA	NA	NA	NA
CASTHMA	2.572772	1.603986	2.522557	1.588256	2.515252	1.585955	2.430928	1.559143	2.369442	1.539299	2.202915	1.484222	2.050653	1.43201
CHD	29.43185	5.425113	26.35377	5.133592	25.92193	5.091359	NA	NA	NA	NA	NA	NA	NA	NA
~~DIABETES~~	33.15382	5.757935	33.09767	5.753058	NA	NA	NA	NA	NA	NA	NA	NA	NA	NA
~~OBESITY~~	3.093372	1.758798	3.070124	1.752177	2.772913	1.665207	2.727348	1.651468	2.361238	1.536632	2.357235	1.535329	2.255838	1.501945
~~STROKE~~	52.04237	7.21404	NA	NA	NA	NA	NA	NA	NA	NA	NA	NA	NA	NA
COPD	13.47433	3.670739	13.12894	3.623388	12.73936	3.569224	7.084034	2.661585	4.404079	2.09859	4.403759	2.098514	2.958887	1.720141
~~KIDNEY~~	47.58153	6.897936	20.7619	4.556523	14.69184	3.832994	7.195956	2.682528	7.126606	2.66957	6.302263	2.510431	NA	NA
TOT_POP_perc_65	1.307825	1.143602	1.305161	1.142437	1.296058	1.138446	1.292535	1.136897	1.279676	1.131228	1.23525	1.111418	1.233578	1.110666

Table 3: Iterative removal of Health Outcome (HO) variables based on adjusted GVIF < 2

2.2 Multicollinearity Analysis and Variable Selection

2.2.1 GLM-1

To address multicollinearity concerns in our health outcome variables, we employed an iterative variable selection process using the Generalized Variance Inflation Factor (GVIF). Table 3 shows the progression of this iterative

Variable	Raw_GVIF	Adjusted_GVIF
factor(urbanicity)	2.017	1.192
lookahead	1.002	1.000
phase	1.113	1.009
model_type	3.471	1.168
mobility_used	3.534	1.371
CASTHMA	20.030	4.475
STROKE	56.230	7.499
DIABETES	73.127	8.551
CHD	73.595	8.579
BPHIGH	27.348	5.230
CANCER	10.058	3.171
OBESITY	5.198	2.280
KIDNEY	78.350	8.852
COPD	56.263	7.501
TOT_POP_perc_65	1.572	1.254

Table 4: GVIF Assessment for GLM-2 (Urbanization Main Effects): Initial Assessment satisfies multicollinearity assumption

process, where variables of interest were systematically removed based on an adjusted GVIF threshold of 2. The adjusted GVIF (A_GVIF) is calculated as $GVIF^{1/(2Df)}$, providing a scale-invariant measure that accounts for the degrees of freedom of each variable.

At the end of this iterative process, our demographic variables of interest (black_perc, hispanic_perc, asian_perc) and data-model characteristic variables (lookahead, phase, model_type, mobility_used) maintained acceptable GVIF values (< 2) throughout the process, supporting their retention in the final model specification. On the other hand, although not strictly necessary [3], the adjusted GVIF values for the control variables (e.g., CASTHMA or COPD) were also below 2. This systematic approach to variable selection helped ensure our model's stability while retaining the most important health outcome controls that showed minimal collinearity with our variables of interest.

2.2.2 GLM-2

Table 4 presents the GVIF assessment for our urbanization model (GLM-2). Most critically, our key variable of interest, urbanization level (factor(urbanicity)), shows a well-contained adjusted GVIF of 1.192, indicating that multicollinearity does not compromise our ability to assess urban-rural disparities. Similarly, all model characteristic variables maintain low adjusted GVIF values during the first iteration: lookahead (1.000), phase (1.009), model_type (1.168), and mobility_used (1.371).

While the health outcome variables exhibit substantial multicollinearity - with *Chronic Kidney Disease* showing the highest adjusted GVIF (8.852), followed by *CHD* (8.579) and *DIABETES* (8.551) - we retain these variables in the model since the coefficients of the variables of interest (urbanization and data-model characteristics) are not affected (their GVIF values are low), and the performance of the control variables as controls is not impaired.

2.3 Model Diagnostics

2.3.1 GLM-1

The diagnostic plots for our main effects model (GLM-1) indicate generally satisfactory model performance while highlighting some limitations that warrant discussion (Figure 3):

Model Fit and Prediction Accuracy: Observed vs. predicted values (Panel A of Figure 3) shows the distribution of observed values at each prediction level through vertical density strips. The dashed line represents perfect prediction (y=x). *Expected*: Short, symmetric vertical distributions centered on the diagonal line with consistent length. *Observed*: Reasonable symmetric clustering around the diagonal at lower values with increasing dispersion at higher predictions, suggesting the model performs better for smaller values of the transformed PBL.

Residual Analysis: Residuals vs. predicted values (Panel B of Figure 3) with zero reference line (dashed red). *Expected*: Random scatter around zero with constant spread. *Observed*: Symmetric pattern around zero with mild heteroscedasticity, showing some variation in spread across predicted values.

Normality Assessment: Q-Q plot of deviance residuals (Panel C of Figure 3) comparing empirical quantiles against theoretical normal distribution. *Expected*: Points following the diagonal line closely if residuals are normally

[3] https://statisticalhorizons.com/multicollinearity/

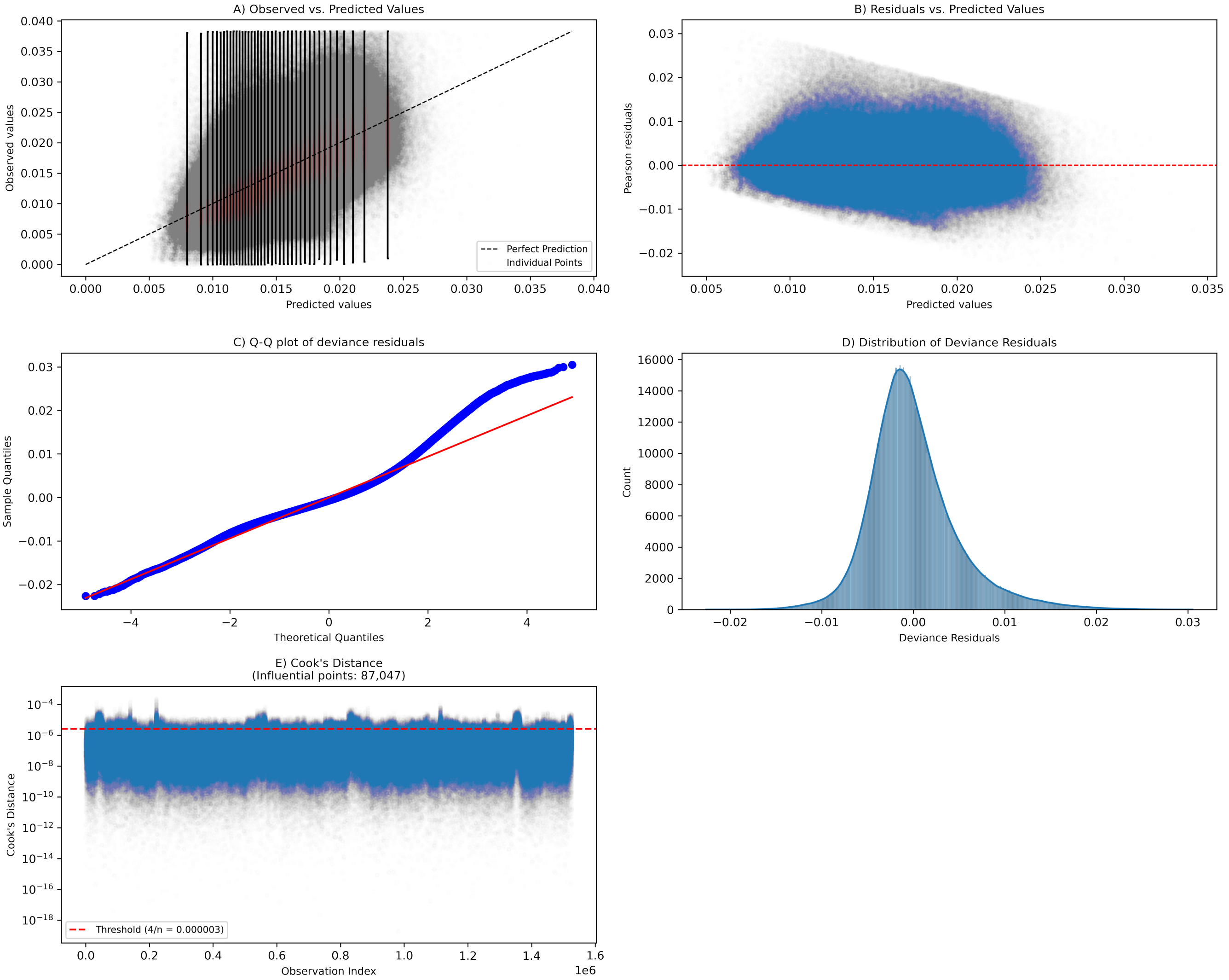

Figure 3: Diagnostic Plots for GLM-1 (Race and Ethnicity Main Effects Model)

distributed. *Observed:* Good alignment in central region with some deviation in tails, indicating approximate normality with slightly heavier tails. Even for the distribution of deviance residuals (Panel D of Figure 3) we see similar patterns of mild heteroscedasticity in the tails. *Expected*: Symmetric, bell-shaped curve centered at zero for normal distribution. *Observed*: Approximately symmetric distribution with slight departure from normality in the tails.

Influence Analysis: Cook's distance plot (Panel E of Figure 3) identifies 87,047 potentially influential points (approximately 5.7% of observations) above the standard threshold of 4/n. While this number might seem high, it is not unexpected given our large sample size (N = 1,526,869) and the complex nature of COVID-19 forecast errors. Further investigation revealed that removing these points did not substantially alter our main findings regarding racial and ethnic disparities.

These diagnostic results collectively suggest that while our model exhibits some departures from ideal GLM assumptions - particularly in terms of residual heteroscedasticity and influential observations - these limitations are reasonably well-managed through our modeling choices (square root transformation and log-link function) and do not compromise the validity of our main conclusions. The symmetric distribution of residuals, reasonable prediction accuracy, and robustness of findings to influential points support the use of this model specification for investigating racial and ethnic disparities in COVID-19 forecasts. Moreover, the observed patterns in the diagnostics are consistent with what we might expect given the inherent challenges in modeling forecast errors across diverse geographic and demographic contexts during a dynamic pandemic. Importantly, sensitivity analyses confirmed that our key findings regarding demographic disparities in forecast accuracy remain stable even when accounting for these model limitations.

These diagnostic results indicate that our model provides a suitable framework for investigating disparities in COVID-19 forecasts, despite some expected limitations. The combination of square root transformation and log-link function helped improve the distributional properties of our dependent variable by reducing right-skewness

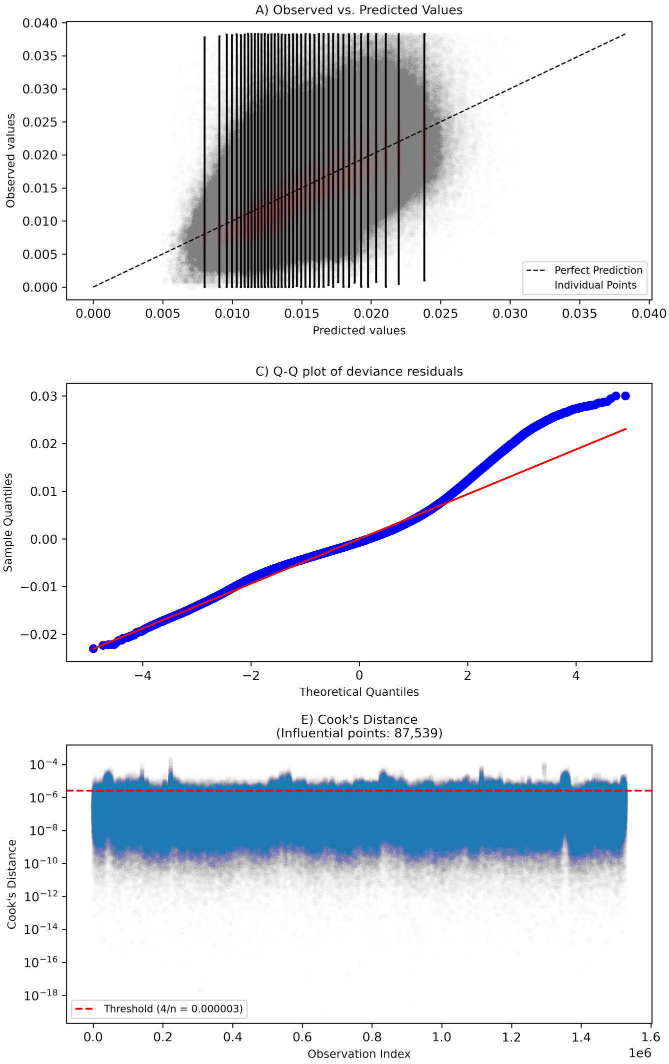

Figure 4: Diagnostic Plots for GLM-2 (Urbanicity Main Effects Model)

and stabilizing variance, although some heteroscedasticity remains visible in the residual plots. The approximate symmetry in residual distribution and generally good alignment in the Q-Q plot's central region suggest that our modeling choices effectively addressed the most severe violations of standard GLM assumptions. While we observe a relatively high number of influential points (5.7% of observations), sensitivity analyses confirmed that our key findings regarding demographic disparities in forecast accuracy remain stable even when these points are excluded.

2.3.2 GLM-2

The diagnostic results for our urbanization model (GLM-2) show patterns highly consistent with those observed in GLM-1, indicating stable model performance across our different specifications (Figure 4):

Model Fit and Prediction Accuracy: Observed vs. predicted values (Panel A) shows the distribution of observed values through vertical density strips, with the dashed line representing perfect prediction. The pattern reveals good prediction accuracy at lower values with increasing dispersion at higher predictions, closely matching what we observed in our race and ethnicity model.

Residual Analysis: The residuals vs. predicted values plot (Panel B) demonstrates symmetric dispersion around zero, with a similar mild heteroscedastic pattern to GLM-1, where the spread of residuals varies somewhat across predicted values.

Normality Assessment: The Q-Q plot (Panel C) and distribution of deviance residuals (Panel D) reveal patterns nearly identical to GLM-1, with good alignment in the central region and slight deviations in the tails. The residual distribution maintains approximate symmetry with minor departures from normality in the extremes.

Influence Analysis: Cook's distance plot (Panel E) identifies 87,539 influential points (5.7% of observations) above the threshold of 4/n, almost identical to GLM-1's 87,047 points. This consistency across models suggests these influential points reflect inherent characteristics of our COVID-19 forecast data rather than model-specific issues.

These diagnostic results reinforce the appropriateness of our modeling approach. The combination of square root transformation and log-link function effectively addressed the main distributional challenges in our data, as evidenced by the similar patterns across both GLM-1 and GLM-2. While some heteroscedasticity remains visible in the residual plots, the consistent symmetric distribution of residuals and robustness to influential points provides confidence in our analyses. The striking similarity between GLM-1 and GLM-2 diagnostics suggests that our model specification is handling both race/ethnicity and urbanization predictors in a stable and reliable manner. This consistency across models strengthens our ability to draw meaningful conclusions about disparities in COVID-19 forecast accuracy across different demographic and geographic contexts.

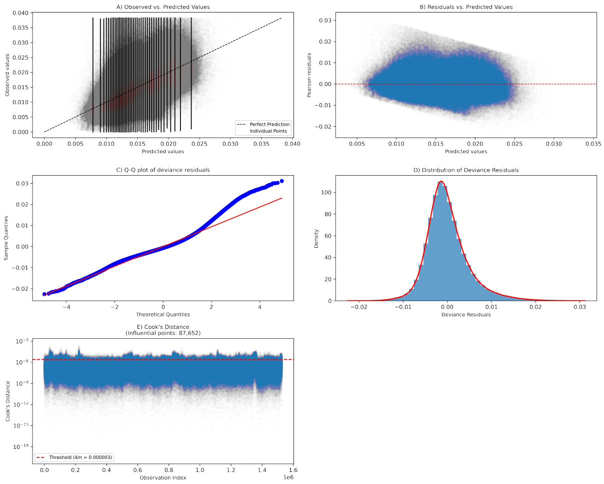

(a) Race and Forecast Lookahead

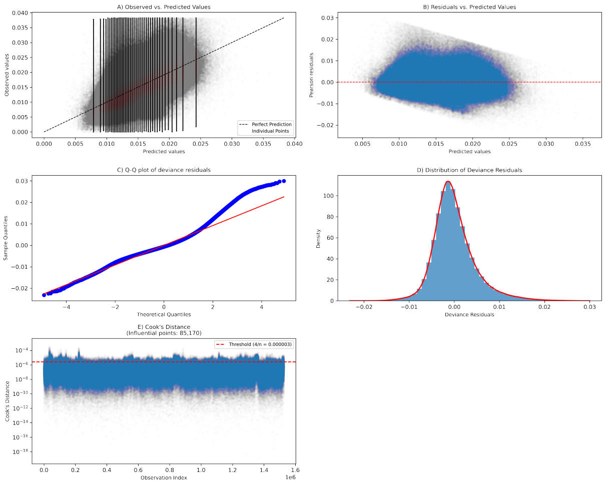

(b) Race and Pandemic Phase

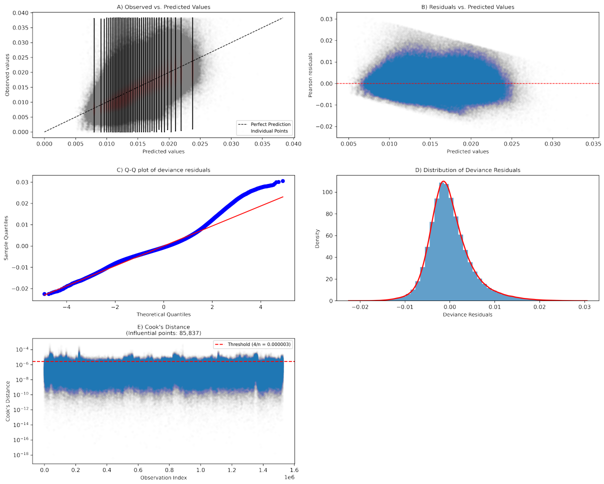

(c) Race and Model Type

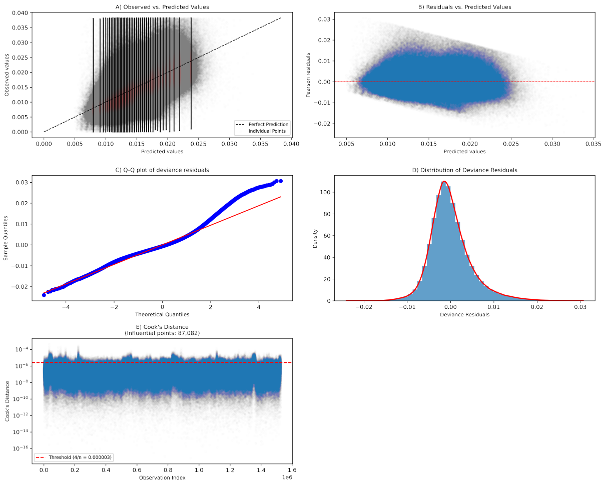

(d) Race and Mobility Usage

Figure 5: Regression diagnostics for the Generalized Linear Models (GLM) analyzing interactions between race/ethnicity and model characteristics: (a) Forecast lookahead interactions (GLM-1a) showing diagnostic plots for models analyzing how prediction horizons (7-28 days) affect racial disparities; (b) Pandemic phase interactions (GLM-1b) presenting diagnostic assessments for models examining how racial disparities vary across different COVID-19 phases; (c) Model type interactions (GLM-1c) showing residual patterns for analyzing racial disparities across different model architectures (compartmental, deep learning, statistical, etc.); and (d) Mobility data usage interactions (GLM-1d) displaying diagnostics for models examining how the incorporation of mobility data impacts prediction fairness across racial groups. Each subplot contains standard regression diagnostic plots: observed vs. fitted values (top left) residuals vs. fitted values (top right), normal Q-Q plot (middle left), scale-location plot (bottom left), and distribution of deviance residuals (bottom right) to validate model assumptions of normality, homoscedasticity, and identify potential influential observations.

State	Coef. (Std. Error)	exp(Coef.)	95% CI	z-value
AK	0.301 (0.004)	1.351	[1.342, 1.361]	84.05***
AL	0.091 (0.003)	1.095	[1.088, 1.101]	28.98***
AR	0.074 (0.003)	1.077	[1.070, 1.084]	22.45***
AZ	0.105 (0.004)	1.111	[1.101, 1.120]	24.81***
CA	0.053 (0.003)	1.054	[1.047, 1.061]	15.53***
CO	0.229 (0.003)	1.257	[1.250, 1.265]	73.51***
DE	0.054 (0.009)	1.055	[1.036, 1.074]	5.87***
GA	0.089 (0.003)	1.094	[1.088, 1.100]	31.55***
HI	0.025 (0.011)	1.025	[1.004, 1.047]	2.32*
IA	0.134 (0.003)	1.144	[1.137, 1.151]	44.51***
ID	0.123 (0.003)	1.131	[1.124, 1.138]	39.78***
IL	0.167 (0.003)	1.182	[1.175, 1.188]	60.01***
IN	0.066 (0.003)	1.068	[1.062, 1.074]	23.28***
KS	0.227 (0.003)	1.255	[1.248, 1.262]	83.67***
KY	0.115 (0.003)	1.122	[1.116, 1.128]	42.27***
LA	0.084 (0.003)	1.088	[1.080, 1.095]	24.90***
MA	-0.019 (0.005)	0.981	[0.970, 0.991]	-3.59***
MD	-0.054 (0.004)	0.947	[0.939, 0.955]	-12.61***
ME	-0.059 (0.005)	0.943	[0.934, 0.952]	-11.88***
MI	0.092 (0.003)	1.096	[1.090, 1.102]	34.04***
MN	0.185 (0.003)	1.203	[1.196, 1.209]	64.50***
MO	0.062 (0.003)	1.064	[1.057, 1.070]	19.54***
MS	0.116 (0.003)	1.123	[1.116, 1.130]	35.47***
MT	0.221 (0.003)	1.247	[1.240, 1.254]	77.02***
NC	0.060 (0.003)	1.062	[1.056, 1.068]	20.19***
ND	0.230 (0.003)	1.258	[1.250, 1.266]	68.57***
NE	0.123 (0.003)	1.131	[1.123, 1.138]	36.15***
NH	0.044 (0.006)	1.045	[1.034, 1.057]	7.79***
NJ	0.035 (0.004)	1.036	[1.027, 1.044]	8.14***
NM	0.041 (0.004)	1.042	[1.035, 1.050]	11.62***
NV	0.037 (0.004)	1.037	[1.028, 1.046]	8.33***
OH	-0.031 (0.003)	0.969	[0.963, 0.975]	-10.21***
OK	0.145 (0.003)	1.156	[1.150, 1.162]	53.63***
OR	-0.007 (0.004)	0.993	[0.986, 1.000]	-1.97*
PA	0.007 (0.003)	1.007	[1.001, 1.013]	2.32*
RI	0.236 (0.007)	1.267	[1.250, 1.284]	34.20***
SC	0.076 (0.004)	1.079	[1.072, 1.087]	21.10***
SD	0.177 (0.003)	1.194	[1.187, 1.201]	56.13***
TN	0.146 (0.003)	1.157	[1.151, 1.164]	50.35***
TX	0.113 (0.003)	1.119	[1.113, 1.125]	39.15***
UT	-0.256 (0.005)	0.774	[0.766, 0.782]	-51.03***
VA	0.075 (0.003)	1.078	[1.072, 1.083]	28.32***
VT	0.022 (0.005)	1.022	[1.012, 1.033]	4.31***
WA	0.046 (0.003)	1.047	[1.040, 1.054]	13.45***
WI	0.179 (0.003)	1.196	[1.189, 1.202]	64.20***
WV	0.055 (0.003)	1.056	[1.050, 1.062]	18.12***
WY	0.184 (0.004)	1.202	[1.194, 1.211]	49.17***

Notes: New York (NY) is the reference state. Significance levels: *** $p < 0.001$, ** $p < 0.01$, * $p < 0.05$. Model uses Gaussian GLM with log link function. Dependent variable is square root of PBL (trimmed).

Table 5: State Fixed Effects for GLM-1 (Reference State: NY)

State	Coef. (Std. Error)	exp(Coef.)	95% CI	z-value
AK	0.181 (0.005)	1.199	[1.188, 1.209]	39.74***
AL	0.076 (0.004)	1.079	[1.071, 1.086]	21.16***
AR	0.030 (0.004)	1.030	[1.022, 1.038]	7.50***
AZ	0.095 (0.005)	1.099	[1.089, 1.110]	18.86***
CA	0.027 (0.004)	1.027	[1.019, 1.034]	7.20***
CO	0.222 (0.004)	1.249	[1.241, 1.258]	62.84***
DE	0.039 (0.009)	1.040	[1.021, 1.059]	4.24***
GA	0.065 (0.004)	1.067	[1.059, 1.074]	18.11***
HI	-0.203 (0.011)	0.816	[0.799, 0.833]	-19.02***
IA	0.106 (0.004)	1.112	[1.104, 1.120]	29.47***
ID	0.091 (0.003)	1.095	[1.088, 1.102]	26.39***
IL	0.149 (0.003)	1.160	[1.153, 1.167]	47.61***
IN	0.085 (0.003)	1.089	[1.082, 1.095]	28.47***
KS	0.183 (0.003)	1.200	[1.193, 1.207]	60.22***
KY	0.148 (0.003)	1.160	[1.153, 1.167]	50.60***
LA	0.081 (0.004)	1.084	[1.076, 1.092]	20.73***
MA	-0.032 (0.005)	0.969	[0.958, 0.979]	-5.80***
MD	-0.069 (0.005)	0.933	[0.925, 0.941]	-15.19***
ME	-0.080 (0.005)	0.924	[0.914, 0.933]	-15.69***
MI	0.070 (0.003)	1.072	[1.066, 1.079]	23.41***
MN	0.115 (0.003)	1.122	[1.114, 1.129]	33.32***
MO	0.062 (0.003)	1.064	[1.057, 1.071]	18.01***
MS	0.077 (0.004)	1.080	[1.072, 1.089]	18.94***
MT	0.172 (0.003)	1.187	[1.179, 1.195]	50.67***
NC	0.031 (0.003)	1.031	[1.024, 1.038]	8.88***
ND	0.142 (0.004)	1.153	[1.143, 1.162]	33.67***
NE	0.071 (0.004)	1.074	[1.065, 1.082]	17.77***
NH	0.035 (0.006)	1.035	[1.024, 1.047]	6.03***
NJ	0.008 (0.005)	1.008	[0.999, 1.017]	1.84
NM	0.041 (0.004)	1.042	[1.034, 1.050]	10.60***
NV	0.020 (0.005)	1.020	[1.011, 1.029]	4.23***
OH	-0.043 (0.003)	0.958	[0.952, 0.964]	-13.39***
OK	0.117 (0.003)	1.124	[1.117, 1.131]	37.54***
OR	-0.032 (0.004)	0.969	[0.962, 0.976]	-8.50***
PA	-0.023 (0.003)	0.977	[0.971, 0.984]	-7.17***
RI	0.246 (0.007)	1.279	[1.261, 1.297]	34.90***
SC	0.059 (0.004)	1.061	[1.053, 1.070]	14.42***
SD	0.083 (0.004)	1.086	[1.077, 1.095]	19.85***
TN	0.167 (0.003)	1.182	[1.175, 1.189]	53.57***
TX	0.127 (0.003)	1.135	[1.128, 1.142]	38.50***
UT	-0.295 (0.005)	0.744	[0.737, 0.752]	-56.03***
VA	0.091 (0.003)	1.096	[1.089, 1.102]	30.99***
VT	-0.020 (0.005)	0.980	[0.970, 0.990]	-3.80***
WA	0.033 (0.004)	1.033	[1.026, 1.041]	9.01***
WI	0.117 (0.003)	1.125	[1.117, 1.132]	35.19***
WV	0.060 (0.004)	1.062	[1.055, 1.069]	17.08***
WY	0.156 (0.004)	1.169	[1.160, 1.178]	38.44***

Notes: New York (NY) is the reference state. Significance levels: *** $p < 0.001$, ** $p < 0.01$, * $p < 0.05$. Model uses Gaussian GLM with log link function. Dependent variable is square root of PBL (trimmed).

Table 6: State Fixed Effects for GLM-2 (Reference State: NY)

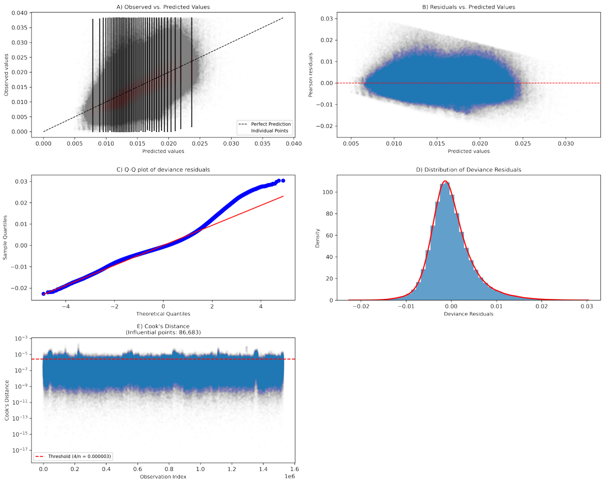

(a) Urbanicity and Lookahead

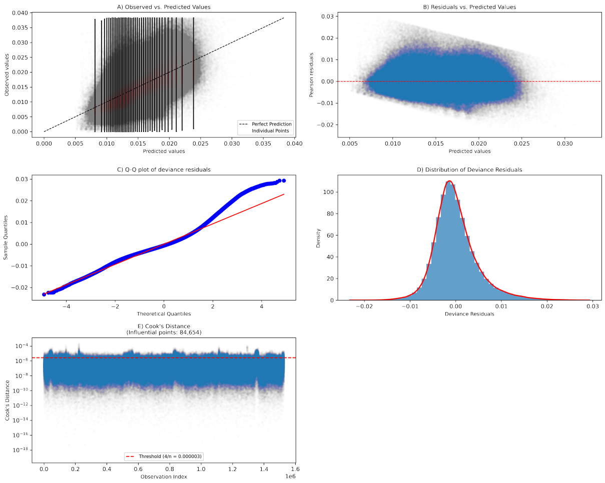

(b) Urbanicity and Pandemic Phase

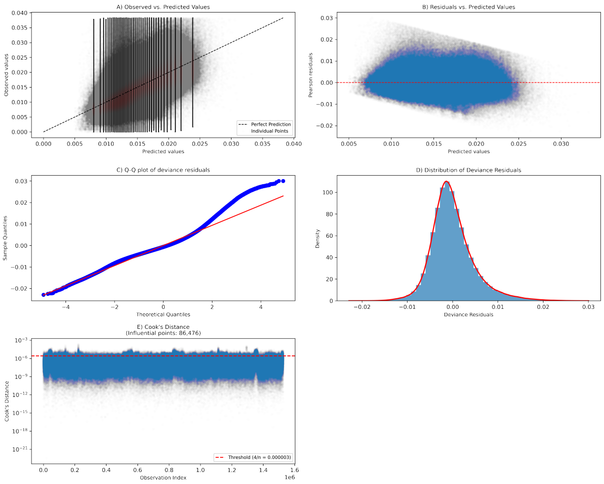

(c) Urbanicity and Model Type

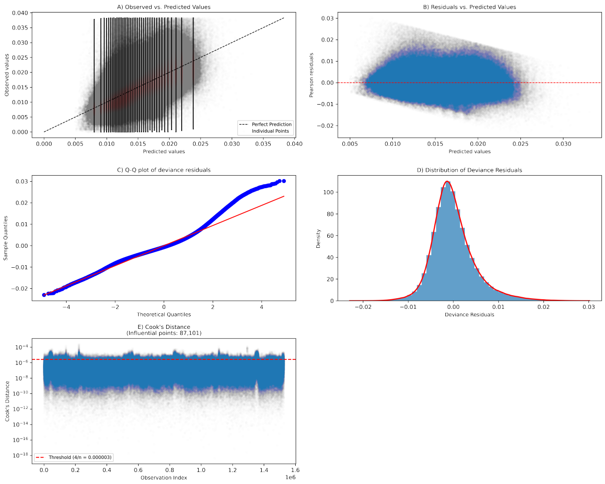

(d) Urbanicity and Mobility Usage

Figure 6: Regression diagnostics for the Generalized Linear Models (GLM) analyzing interactions between urbanicity and model characteristics: (a) Forecast lookahead interactions (GLM-1a) showing diagnostic plots for models analyzing how prediction horizons (7-28 days) affect racial disparities; (b) Pandemic phase interactions (GLM-1b) presenting diagnostic assessments for models examining how racial disparities vary across different COVID-19 phases; (c) Model type interactions (GLM-1c) showing residual patterns for analyzing racial disparities across different model architectures (compartmental, deep learning, statistical, etc.); and (d) Mobility data usage interactions (GLM-1d) displaying diagnostics for models examining how the incorporation of mobility data impacts prediction fairness across racial groups. Each subplot contains standard regression diagnostic plots: observed vs. fitted values (top left) residuals vs. fitted values (top right), normal Q-Q plot (middle left), scale-location plot (bottom left), and distribution of deviance residuals (bottom right) to validate model assumptions of normality, homoscedasticity, and identify potential influential observations.

Variable	Coef. (Std. Error)	exp(Coef.)	95% CI	z-value
Lookahead (ref: 7-day)				
14-day ahead	0.108 (0.001)	1.114	[1.111, 1.116]	93.23***
21-day ahead	0.183 (0.001)	1.201	[1.199, 1.204]	162.93***
28-day ahead	0.251 (0.001)	1.285	[1.283, 1.288]	230.21***
Model Type (ref: Compartmental)				
Baseline Models	0.114 (0.002)	1.121	[1.118, 1.124]	73.02***
Deep Learning	0.037 (0.001)	1.037	[1.035, 1.039]	40.15***
Ensemble	0.007 (0.001)	1.007	[1.005, 1.009]	7.97***
Statistical	0.073 (0.001)	1.076	[1.074, 1.078]	89.84***
Mobility Used (ref: No)				
Mixed	-0.146 (0.001)	0.864	[0.862, 0.866]	-124.31***
Yes	-0.004 (0.001)	0.996	[0.994, 0.997]	-5.55***
Phase (ref: Phase 0)				
Phase 1	0.368 (0.001)	1.445	[1.442, 1.448]	377.45***
Phase 2	-0.116 (0.001)	0.891	[0.888, 0.893]	-99.57***
Phase 3	0.251 (0.001)	1.285	[1.283, 1.288]	236.62***
Phase 4	0.440 (0.001)	1.553	[1.550, 1.556]	417.08***
Phase 5	-0.031 (0.001)	0.969	[0.967, 0.972]	-22.76***
Phase 6	0.141 (0.001)	1.152	[1.149, 1.154]	112.44***
Health Outcomes				
Asthma	-0.041 (0.001)	0.959	[0.958, 0.961]	-43.32***
Obesity	0.003 (0.0001)	1.003	[1.003, 1.004]	25.28***
COPD	0.045 (0.0005)	1.046	[1.045, 1.047]	92.63***
Age 65+	0.190 (0.007)	1.209	[1.193, 1.225]	28.05***
Intercept	-4.686 (0.009)	0.009	[0.009, 0.009]	-508.57***

Notes: Race × Lookahead interactions shown in main manuscript table. Significance levels: *** $p < 0.001$, ** $p < 0.01$, * $p < 0.05$. Model uses Gaussian GLM with log link function. Dependent variable is square root of PBL.

Table 7: Main Effects for GLM-1a (Race × Lookahead Interactions)

State	Coef. (Std. Error)	exp(Coef.)	95% CI	z-value
AK	0.299 (0.004)	1.349	[1.339, 1.358]	83.52***
AL	0.091 (0.003)	1.095	[1.088, 1.102]	29.04***
AR	0.075 (0.003)	1.077	[1.070, 1.084]	22.65***
AZ	0.104 (0.004)	1.110	[1.101, 1.119]	24.70***
CA	0.054 (0.003)	1.056	[1.049, 1.063]	16.03***
CO	0.228 (0.003)	1.256	[1.248, 1.263]	73.17***
DE	0.054 (0.009)	1.056	[1.037, 1.075]	5.96***
GA	0.090 (0.003)	1.094	[1.088, 1.100]	31.60***
HI	0.020 (0.011)	1.020	[0.999, 1.042]	1.84
IA	0.135 (0.003)	1.144	[1.138, 1.151]	44.69***
ID	0.123 (0.003)	1.131	[1.124, 1.138]	39.64***
IL	0.167 (0.003)	1.182	[1.175, 1.188]	60.12***
IN	0.066 (0.003)	1.068	[1.062, 1.074]	23.35***
KS	0.227 (0.003)	1.255	[1.249, 1.262]	83.85***
KY	0.116 (0.003)	1.123	[1.117, 1.129]	42.39***
LA	0.084 (0.003)	1.088	[1.081, 1.095]	25.02***
MA	-0.019 (0.005)	0.981	[0.971, 0.991]	-3.57***
MD	-0.054 (0.004)	0.948	[0.940, 0.956]	-12.48***
ME	-0.059 (0.005)	0.942	[0.933, 0.951]	-12.05***
MI	0.092 (0.003)	1.096	[1.090, 1.102]	34.01***
MN	0.185 (0.003)	1.203	[1.196, 1.210]	64.60***
MO	0.062 (0.003)	1.064	[1.058, 1.071]	19.79***
MS	0.116 (0.003)	1.123	[1.116, 1.130]	35.51***
MT	0.220 (0.003)	1.246	[1.239, 1.253]	76.92***
NC	0.061 (0.003)	1.062	[1.056, 1.069]	20.31***
ND	0.230 (0.003)	1.259	[1.250, 1.267]	68.76***
NE	0.123 (0.003)	1.131	[1.124, 1.139]	36.40***
NH	0.044 (0.006)	1.045	[1.033, 1.056]	7.73***
NJ	0.036 (0.004)	1.037	[1.028, 1.046]	8.45***
NM	0.041 (0.004)	1.042	[1.034, 1.049]	11.49***
NV	0.037 (0.004)	1.038	[1.029, 1.047]	8.50***
OH	-0.031 (0.003)	0.970	[0.964, 0.976]	-10.03***
OK	0.145 (0.003)	1.156	[1.150, 1.162]	53.68***
OR	-0.007 (0.004)	0.993	[0.986, 1.000]	-2.05*
PA	0.007 (0.003)	1.007	[1.001, 1.013]	2.34*
RI	0.236 (0.007)	1.266	[1.249, 1.283]	34.10***
SC	0.077 (0.004)	1.080	[1.072, 1.087]	21.21***
SD	0.178 (0.003)	1.194	[1.187, 1.202]	56.27***
TN	0.146 (0.003)	1.158	[1.151, 1.164]	50.44***
TX	0.113 (0.003)	1.120	[1.113, 1.126]	39.37***
UT	-0.257 (0.005)	0.774	[0.766, 0.781]	-51.19***
VA	0.075 (0.003)	1.078	[1.072, 1.083]	28.39***
VT	0.021 (0.005)	1.021	[1.011, 1.031]	4.11***
WA	0.045 (0.003)	1.047	[1.040, 1.054]	13.35***
WI	0.178 (0.003)	1.195	[1.189, 1.202]	64.17***
WV	0.055 (0.003)	1.057	[1.050, 1.063]	18.24***
WY	0.184 (0.004)	1.202	[1.193, 1.211]	49.10***

Notes: New York (NY) is the reference state. Significance levels: *** $p < 0.001$, ** $p < 0.01$, * $p < 0.05$. Model uses Gaussian GLM with log link function. Dependent variable is square root of PBL.

Table 8: State Fixed Effects for GLM-1a (Reference State: NY)

Variable	Coef. (Std. Error)	exp(Coef.)	95% CI	z-value
Lookahead (ref: 7-day)				
14-day ahead	0.112 (0.001)	1.119	[1.117, 1.121]	146.51***
21-day ahead	0.191 (0.001)	1.210	[1.208, 1.212]	254.30***
28-day ahead	0.261 (0.001)	1.299	[1.297, 1.301]	359.63***
Model Type (ref: Compartmental)				
Baseline Models	0.113 (0.002)	1.120	[1.117, 1.123]	74.07***
Deep Learning	0.036 (0.001)	1.037	[1.035, 1.039]	40.93***
Ensemble	0.007 (0.001)	1.007	[1.005, 1.008]	7.71***
Statistical	0.072 (0.001)	1.074	[1.073, 1.076]	89.95***
Mobility Used (ref: No)				
Not Applicable	-0.146 (0.001)	0.864	[0.862, 0.866]	-126.55***
Yes	-0.005 (0.001)	0.995	[0.994, 0.997]	-6.17***
Phase (ref: Phase 0)				
Phase 1	0.481 (0.001)	1.618	[1.613, 1.623]	328.46***
Phase 2	0.009 (0.002)	1.009	[1.006, 1.013]	5.29***
Phase 3	0.373 (0.002)	1.453	[1.448, 1.457]	235.62***
Phase 4	0.618 (0.002)	1.856	[1.850, 1.862]	401.03***
Phase 5	0.090 (0.002)	1.094	[1.090, 1.098]	45.57***
Phase 6	0.252 (0.002)	1.286	[1.282, 1.291]	136.28***
Health Outcomes				
Asthma	-0.041 (0.001)	0.960	[0.958, 0.962]	-43.72***
Obesity	0.003 (0.0001)	1.003	[1.003, 1.003]	22.83***
COPD	0.044 (0.0005)	1.045	[1.044, 1.046]	93.22***
Age 65+	0.156 (0.007)	1.169	[1.154, 1.184]	23.51***
Intercept	-4.779 (0.009)	0.008	[0.008, 0.009]	-528.25***

Notes: Race × Phase interactions shown in main manuscript table. Significance levels: *** $p < 0.001$, ** $p < 0.01$, * $p < 0.05$. Model uses Gaussian GLM with log link function. Dependent variable is square root of PBL.

Table 9: Main Effects for GLM-1b (Race × Phase Interactions)

State	Coef. (Std. Error)	exp(Coef.)	95% CI	z-value
AK	0.290 (0.004)	1.337	[1.328, 1.346]	82.71***
AL	0.084 (0.003)	1.088	[1.082, 1.095]	27.64***
AR	0.071 (0.003)	1.073	[1.066, 1.080]	21.95***
AZ	0.099 (0.004)	1.104	[1.095, 1.112]	23.86***
CA	0.044 (0.003)	1.045	[1.039, 1.052]	13.38***
CO	0.217 (0.003)	1.242	[1.235, 1.250]	71.21***
DE	0.052 (0.009)	1.054	[1.035, 1.072]	5.82***
GA	0.089 (0.003)	1.093	[1.087, 1.099]	32.08***
HI	0.002 (0.010)	1.002	[0.982, 1.023]	0.21
IA	0.125 (0.003)	1.133	[1.127, 1.140]	42.33***
ID	0.112 (0.003)	1.119	[1.112, 1.125]	37.06***
IL	0.160 (0.003)	1.173	[1.167, 1.180]	58.96***
IN	0.058 (0.003)	1.060	[1.054, 1.066]	21.23***
KS	0.221 (0.003)	1.248	[1.241, 1.254]	83.66***
KY	0.109 (0.003)	1.116	[1.110, 1.122]	41.13***
LA	0.088 (0.003)	1.092	[1.085, 1.099]	26.87***
MA	-0.017 (0.005)	0.983	[0.973, 0.993]	-3.28**
MD	-0.059 (0.004)	0.943	[0.935, 0.951]	-13.91***
ME	-0.061 (0.005)	0.941	[0.932, 0.950]	-12.76***
MI	0.088 (0.003)	1.092	[1.086, 1.097]	33.35***
MN	0.179 (0.003)	1.196	[1.189, 1.203]	64.10***
MO	0.055 (0.003)	1.057	[1.051, 1.063]	17.94***
MS	0.112 (0.003)	1.118	[1.111, 1.125]	35.08***
MT	0.209 (0.003)	1.232	[1.225, 1.239]	74.63***
NC	0.056 (0.003)	1.057	[1.051, 1.063]	19.11***
ND	0.219 (0.003)	1.245	[1.237, 1.253]	66.95***
NE	0.116 (0.003)	1.123	[1.116, 1.131]	35.04***
NH	0.047 (0.005)	1.048	[1.037, 1.060]	8.64***
NJ	0.041 (0.004)	1.042	[1.033, 1.051]	9.80***
NM	0.023 (0.003)	1.023	[1.016, 1.030]	6.53***
NV	0.033 (0.004)	1.033	[1.025, 1.042]	7.67***
OH	-0.035 (0.003)	0.965	[0.960, 0.971]	-11.88***
OK	0.138 (0.003)	1.148	[1.142, 1.154]	52.25***
OR	-0.013 (0.003)	0.987	[0.980, 0.994]	-3.82***
PA	0.004 (0.003)	1.004	[0.998, 1.009]	1.27
RI	0.233 (0.007)	1.262	[1.246, 1.279]	34.62***
SC	0.077 (0.004)	1.081	[1.073, 1.088]	21.92***
SD	0.167 (0.003)	1.181	[1.174, 1.189]	54.02***
TN	0.138 (0.003)	1.148	[1.142, 1.155]	48.83***
TX	0.110 (0.003)	1.116	[1.110, 1.122]	39.11***
UT	-0.268 (0.005)	0.765	[0.757, 0.772]	-54.66***
VA	0.071 (0.003)	1.073	[1.068, 1.079]	27.49***
VT	0.020 (0.005)	1.020	[1.010, 1.030]	4.02***
WA	0.040 (0.003)	1.040	[1.034, 1.047]	11.89***
WI	0.170 (0.003)	1.185	[1.178, 1.191]	62.46***
WV	0.051 (0.003)	1.052	[1.046, 1.058]	17.29***
WY	0.173 (0.004)	1.189	[1.181, 1.198]	47.32***

Notes: New York (NY) is the reference state. Significance levels: *** $p < 0.001$, ** $p < 0.01$, * $p < 0.05$. Model uses Gaussian GLM with log link function. Dependent variable is square root of PBL.

Table 10: State Fixed Effects for GLM-1b (Reference State: NY)

Variable	Coef. (Std. Error)	exp(Coef.)	95% CI	z-value
Lookahead (ref: 7-day)				
14-day ahead	0.113 (0.001)	1.119	[1.117, 1.121]	143.58***
21-day ahead	0.191 (0.001)	1.211	[1.209, 1.213]	249.41***
28-day ahead	0.262 (0.001)	1.300	[1.298, 1.302]	352.74***
Model Type (ref: Compartmental)				
Baseline Models	0.103 (0.002)	1.109	[1.104, 1.113]	51.37***
Deep Learning	0.044 (0.001)	1.045	[1.042, 1.048]	33.29***
Ensemble	0.014 (0.001)	1.014	[1.012, 1.016]	12.54***
Statistical	0.080 (0.001)	1.084	[1.081, 1.086]	70.34***
Mobility Used (ref: No)				
Not Applicable	-0.147 (0.001)	0.864	[0.862, 0.866]	-124.45***
Yes	-0.005 (0.001)	0.996	[0.994, 0.997]	-5.90***
Phase (ref: Phase 0)				
Phase 1	0.368 (0.001)	1.445	[1.442, 1.447]	376.90***
Phase 2	-0.116 (0.001)	0.890	[0.888, 0.892]	-99.91***
Phase 3	0.251 (0.001)	1.285	[1.282, 1.288]	236.23***
Phase 4	0.440 (0.001)	1.552	[1.549, 1.556]	416.68***
Phase 5	-0.031 (0.001)	0.969	[0.967, 0.972]	-22.96***
Phase 6	0.141 (0.001)	1.151	[1.148, 1.154]	112.17***
Health Outcomes				
Asthma	-0.042 (0.001)	0.959	[0.957, 0.961]	-43.77***
Obesity	0.003 (0.0001)	1.003	[1.003, 1.004]	25.92***
COPD	0.045 (0.0005)	1.046	[1.045, 1.047]	93.07***
Age 65+	0.194 (0.007)	1.214	[1.199, 1.231]	28.74***
Intercept	-4.697 (0.009)	0.009	[0.009, 0.009]	-510.25***

Notes: Race × Model Type interactions shown in main manuscript table. Significance levels: *** $p < 0.001$, ** $p < 0.01$, * $p < 0.05$. Model uses Gaussian GLM with log link function. Dependent variable is square root of PBL.

Table 11: Main Effects for GLM-1c (Race × Model Type Interactions)

State	Coef. (Std. Error)	exp(Coef.)	95% CI	z-value
AK	0.301 (0.004)	1.351	[1.342, 1.361]	84.05***
AL	0.090 (0.003)	1.095	[1.088, 1.101]	28.98***
AR	0.074 (0.003)	1.077	[1.070, 1.084]	22.44***
AZ	0.105 (0.004)	1.110	[1.101, 1.120]	24.80***
CA	0.053 (0.003)	1.054	[1.047, 1.062]	15.62***
CO	0.229 (0.003)	1.258	[1.250, 1.265]	73.56***
DE	0.054 (0.009)	1.055	[1.036, 1.074]	5.85***
GA	0.089 (0.003)	1.093	[1.087, 1.100]	31.52***
HI	0.025 (0.011)	1.025	[1.004, 1.047]	2.31*
IA	0.134 (0.003)	1.144	[1.137, 1.150]	44.46***
ID	0.123 (0.003)	1.131	[1.124, 1.138]	39.72***
IL	0.167 (0.003)	1.181	[1.175, 1.188]	59.98***
IN	0.065 (0.003)	1.068	[1.062, 1.074]	23.24***
KS	0.227 (0.003)	1.255	[1.248, 1.261]	83.65***
KY	0.115 (0.003)	1.122	[1.116, 1.128]	42.23***
LA	0.084 (0.003)	1.087	[1.080, 1.095]	24.83***
MA	-0.019 (0.005)	0.981	[0.970, 0.991]	-3.58***
MD	-0.055 (0.004)	0.947	[0.939, 0.955]	-12.68***
ME	-0.059 (0.005)	0.943	[0.934, 0.952]	-11.92***
MI	0.092 (0.003)	1.096	[1.090, 1.102]	33.99***
MN	0.184 (0.003)	1.202	[1.196, 1.209]	64.44***
MO	0.062 (0.003)	1.064	[1.057, 1.070]	19.50***
MS	0.116 (0.003)	1.123	[1.116, 1.130]	35.46***
MT	0.220 (0.003)	1.247	[1.240, 1.254]	76.96***
NC	0.060 (0.003)	1.062	[1.056, 1.068]	20.16***
ND	0.229 (0.003)	1.258	[1.249, 1.266]	68.51***
NE	0.123 (0.003)	1.130	[1.123, 1.138]	36.15***
NH	0.044 (0.006)	1.045	[1.033, 1.057]	7.77***
NJ	0.035 (0.004)	1.036	[1.027, 1.044]	8.14***
NM	0.041 (0.004)	1.042	[1.035, 1.049]	11.56***
NV	0.036 (0.004)	1.037	[1.028, 1.046]	8.29***
OH	-0.031 (0.003)	0.969	[0.963, 0.975]	-10.25***
OK	0.145 (0.003)	1.156	[1.150, 1.162]	53.60***
OR	-0.007 (0.004)	0.993	[0.986, 1.000]	-2.03*
PA	0.007 (0.003)	1.007	[1.001, 1.013]	2.27*
RI	0.236 (0.007)	1.267	[1.249, 1.284]	34.18***
SC	0.076 (0.004)	1.079	[1.072, 1.087]	21.08***
SD	0.177 (0.003)	1.194	[1.186, 1.201]	56.09***
TN	0.146 (0.003)	1.157	[1.151, 1.164]	50.32***
TX	0.113 (0.003)	1.119	[1.113, 1.125]	39.16***
UT	-0.256 (0.005)	0.774	[0.766, 0.782]	-51.05***
VA	0.075 (0.003)	1.077	[1.072, 1.083]	28.24***
VT	0.022 (0.005)	1.022	[1.012, 1.032]	4.30***
WA	0.046 (0.003)	1.047	[1.040, 1.054]	13.36***
WI	0.178 (0.003)	1.195	[1.189, 1.202]	64.15***
WV	0.055 (0.003)	1.056	[1.050, 1.062]	18.08***
WY	0.184 (0.004)	1.202	[1.193, 1.211]	49.13***

Notes: New York (NY) is the reference state. Significance levels: *** $p < 0.001$, ** $p < 0.01$, * $p < 0.05$. Model uses Gaussian GLM with log link function. Dependent variable is square root of PBL.

Table 12: State Fixed Effects for GLM-1c (Reference State: NY)

Variable	Coef. (Std. Error)	exp(Coef.)	95% CI	z-value
Lookahead (ref: 7-day)				
14-day ahead	0.113 (0.001)	1.119	[1.118, 1.121]	143.64***
21-day ahead	0.191 (0.001)	1.211	[1.209, 1.213]	249.53***
28-day ahead	0.262 (0.001)	1.300	[1.298, 1.302]	352.84***
Model Type (ref: Compartmental)				
Baseline Models	0.114 (0.002)	1.121	[1.118, 1.125]	73.05***
Deep Learning	0.037 (0.001)	1.037	[1.036, 1.039]	40.30***
Ensemble	0.007 (0.001)	1.007	[1.005, 1.009]	8.11***
Statistical	0.073 (0.001)	1.076	[1.074, 1.077]	89.57***
Mobility Used (ref: No)				
Not Applicable	-0.137 (0.001)	0.872	[0.870, 0.875]	-92.95***
Yes	0.010 (0.001)	1.010	[1.008, 1.012]	9.76***
Phase (ref: Phase 0)				
Phase 1	0.368 (0.001)	1.444	[1.442, 1.447]	376.83***
Phase 2	-0.117 (0.001)	0.890	[0.888, 0.892]	-100.06***
Phase 3	0.250 (0.001)	1.285	[1.282, 1.287]	236.15***
Phase 4	0.440 (0.001)	1.552	[1.549, 1.555]	416.73***
Phase 5	-0.031 (0.001)	0.969	[0.967, 0.972]	-22.94***
Phase 6	0.141 (0.001)	1.151	[1.148, 1.154]	111.97***
Health Outcomes				
Asthma	-0.042 (0.001)	0.959	[0.957, 0.961]	-43.86***
Obesity	0.003 (0.0001)	1.003	[1.003, 1.004]	25.84***
COPD	0.045 (0.0005)	1.046	[1.045, 1.047]	93.25***
Age 65+	0.194 (0.007)	1.214	[1.198, 1.230]	28.63***
Intercept	-4.704 (0.009)	0.009	[0.009, 0.009]	-510.35***

Notes: Race × Mobility interactions shown in main manuscript table. Significance levels: *** $p < 0.001$, ** $p < 0.01$, * $p < 0.05$. Model uses Gaussian GLM with log link function. Dependent variable is square root of PBL.

Table 13: Main Effects for GLM-1d (Race × Mobility Interactions)

State	Coef. (Std. Error)	exp(Coef.)	95% CI	z-value
AK	0.301 (0.004)	1.351	[1.342, 1.361]	84.17***
AL	0.090 (0.003)	1.094	[1.088, 1.101]	28.83***
AR	0.074 (0.003)	1.076	[1.069, 1.083]	22.34***
AZ	0.104 (0.004)	1.110	[1.101, 1.119]	24.68***
CA	0.052 (0.003)	1.053	[1.046, 1.060]	15.22***
CO	0.229 (0.003)	1.257	[1.249, 1.265]	73.44***
DE	0.053 (0.009)	1.055	[1.036, 1.074]	5.83***
GA	0.089 (0.003)	1.093	[1.087, 1.099]	31.48***
HI	0.027 (0.011)	1.028	[1.006, 1.049]	2.55*
IA	0.134 (0.003)	1.143	[1.136, 1.150]	44.32***
ID	0.123 (0.003)	1.131	[1.124, 1.138]	39.69***
IL	0.166 (0.003)	1.181	[1.175, 1.188]	59.90***
IN	0.065 (0.003)	1.067	[1.061, 1.073]	23.14***
KS	0.227 (0.003)	1.254	[1.248, 1.261]	83.62***
KY	0.115 (0.003)	1.122	[1.116, 1.128]	42.13***
LA	0.084 (0.003)	1.087	[1.080, 1.094]	24.82***
MA	-0.019 (0.005)	0.981	[0.971, 0.992]	-3.48***
MD	-0.055 (0.004)	0.947	[0.939, 0.955]	-12.70***
ME	-0.058 (0.005)	0.943	[0.934, 0.952]	-11.86***
MI	0.091 (0.003)	1.096	[1.090, 1.102]	33.96***
MN	0.184 (0.003)	1.202	[1.196, 1.209]	64.44***
MO	0.061 (0.003)	1.063	[1.057, 1.070]	19.37***
MS	0.116 (0.003)	1.123	[1.116, 1.130]	35.42***
MT	0.220 (0.003)	1.246	[1.239, 1.253]	76.88***
NC	0.060 (0.003)	1.062	[1.056, 1.068]	20.10***
ND	0.229 (0.003)	1.257	[1.249, 1.266]	68.42***
NE	0.122 (0.003)	1.130	[1.122, 1.138]	36.02***
NH	0.044 (0.006)	1.045	[1.034, 1.057]	7.85***
NJ	0.035 (0.004)	1.036	[1.027, 1.045]	8.20***
NM	0.041 (0.004)	1.041	[1.034, 1.049]	11.41***
NV	0.036 (0.004)	1.037	[1.028, 1.046]	8.27***
OH	-0.032 (0.003)	0.969	[0.963, 0.975]	-10.33***
OK	0.145 (0.003)	1.156	[1.150, 1.162]	53.51***
OR	-0.007 (0.004)	0.993	[0.986, 1.000]	-2.00*
PA	0.007 (0.003)	1.007	[1.001, 1.013]	2.28*
RI	0.236 (0.007)	1.267	[1.250, 1.284]	34.19***
SC	0.076 (0.004)	1.079	[1.072, 1.087]	21.11***
SD	0.177 (0.003)	1.193	[1.186, 1.201]	55.94***
TN	0.146 (0.003)	1.157	[1.150, 1.163]	50.20***
TX	0.112 (0.003)	1.119	[1.113, 1.125]	39.08***
UT	-0.257 (0.005)	0.773	[0.766, 0.781]	-51.24***
VA	0.075 (0.003)	1.077	[1.072, 1.083]	28.23***
VT	0.022 (0.005)	1.022	[1.012, 1.033]	4.32***
WA	0.046 (0.003)	1.047	[1.040, 1.054]	13.48***
WI	0.178 (0.003)	1.195	[1.189, 1.202]	64.13***
WV	0.054 (0.003)	1.056	[1.050, 1.062]	18.03***
WY	0.184 (0.004)	1.202	[1.193, 1.211]	49.07***

Notes: New York (NY) is the reference state. Significance levels: *** $p < 0.001$, ** $p < 0.01$, * $p < 0.05$. Model uses Gaussian GLM with log link function. Dependent variable is square root of PBL.

Table 14: State Fixed Effects for GLM-1d (Reference State: NY)

**Variable**	**Coef. (Std. Error)**	**exp(Coef.)**	**95% CI**	**z-value**
Lookahead (ref: 7-day)				
14-day ahead	0.142 (0.002)	1.152	[1.147, 1.157]	58.71***
21-day ahead	0.249 (0.002)	1.283	[1.277, 1.288]	106.78***
28-day ahead	0.340 (0.002)	1.404	[1.398, 1.411]	151.23***
Model Type (ref: Compartmental)				
Baseline Models	0.114 (0.002)	1.121	[1.118, 1.125]	73.19***
Deep Learning	0.037 (0.001)	1.037	[1.035, 1.039]	40.18***
Ensemble	0.007 (0.001)	1.007	[1.005, 1.009]	8.03***
Statistical	0.073 (0.001)	1.076	[1.074, 1.078]	90.13***
Mobility Used (ref: No)				
Not Applicable	-0.147 (0.001)	0.864	[0.862, 0.866]	-124.59***
Yes	-0.004 (0.001)	0.996	[0.994, 0.997]	-5.40***
Phase (ref: Phase 0)				
Phase 1	0.368 (0.001)	1.445	[1.442, 1.448]	377.92***
Phase 2	-0.116 (0.001)	0.890	[0.888, 0.892]	-100.11***
Phase 3	0.250 (0.001)	1.285	[1.282, 1.287]	236.51***
Phase 4	0.440 (0.001)	1.552	[1.549, 1.555]	417.30***
Phase 5	-0.031 (0.001)	0.969	[0.967, 0.972]	-22.76***
Phase 6	0.141 (0.001)	1.151	[1.148, 1.154]	112.47***
Health Outcomes				
Asthma	-0.045 (0.001)	0.956	[0.954, 0.958]	-34.87***
Obesity	0.004 (0.0001)	1.004	[1.004, 1.005]	31.91***
High Blood Pressure	-0.002 (0.0003)	0.998	[0.997, 0.998]	-7.73***
COPD	-0.000 (0.001)	1.000	[0.998, 1.002]	-0.01
Stroke	-0.019 (0.003)	0.981	[0.974, 0.987]	-5.65***
Cancer	-0.016 (0.003)	0.984	[0.978, 0.989]	-5.74***
Coronary Heart Disease	0.085 (0.003)	1.089	[1.084, 1.094]	33.35***
Diabetes	-0.005 (0.001)	0.995	[0.993, 0.997]	-5.34***
Kidney Disease	0.042 (0.006)	1.043	[1.030, 1.056]	6.56***
Age 65+	0.106 (0.007)	1.112	[1.097, 1.127]	15.48***
Intercept	-4.755 (0.018)	0.009	[0.008, 0.009]	-264.84***

Notes: Urbanicity × Lookahead interactions shown in main manuscript table. Significance levels: *** $p < 0.001$, ** $p < 0.01$, * $p < 0.05$. Model uses Gaussian GLM with log link function. Dependent variable is square root of PBL.

Table 15: Main Effects for GLM-2a (Urbanicity × Lookahead Interactions)

State	Coef. (Std. Error)	exp(Coef.)	95% CI	z-value
AK	0.181 (0.005)	1.199	[1.188, 1.210]	39.86***
AL	0.076 (0.004)	1.079	[1.071, 1.086]	21.19***
AR	0.030 (0.004)	1.030	[1.022, 1.038]	7.53***
AZ	0.095 (0.005)	1.099	[1.089, 1.110]	18.88***
CA	0.027 (0.004)	1.027	[1.020, 1.035]	7.34***
CO	0.222 (0.004)	1.249	[1.240, 1.257]	62.85***
DE	0.039 (0.009)	1.040	[1.021, 1.059]	4.27***
GA	0.064 (0.004)	1.067	[1.059, 1.074]	18.10***
HI	-0.204 (0.011)	0.816	[0.799, 0.833]	-19.09***
IA	0.106 (0.004)	1.112	[1.104, 1.120]	29.51***
ID	0.091 (0.003)	1.095	[1.088, 1.102]	26.42***
IL	0.148 (0.003)	1.160	[1.153, 1.167]	47.64***
IN	0.085 (0.003)	1.088	[1.082, 1.095]	28.46***
KS	0.183 (0.003)	1.200	[1.193, 1.208]	60.35***
KY	0.148 (0.003)	1.160	[1.153, 1.166]	50.66***
LA	0.081 (0.004)	1.084	[1.076, 1.092]	20.76***
MA	-0.031 (0.005)	0.969	[0.959, 0.980]	-5.73***
MD	-0.069 (0.005)	0.933	[0.925, 0.942]	-15.16***
ME	-0.080 (0.005)	0.924	[0.914, 0.933]	-15.71***
MI	0.070 (0.003)	1.072	[1.066, 1.079]	23.46***
MN	0.115 (0.003)	1.122	[1.114, 1.129]	33.39***
MO	0.062 (0.003)	1.064	[1.057, 1.071]	18.06***
MS	0.077 (0.004)	1.080	[1.072, 1.089]	18.97***
MT	0.172 (0.003)	1.187	[1.179, 1.195]	50.77***
NC	0.031 (0.003)	1.031	[1.024, 1.038]	8.87***
ND	0.142 (0.004)	1.153	[1.143, 1.163]	33.80***
NE	0.071 (0.004)	1.074	[1.066, 1.082]	17.89***
NH	0.034 (0.006)	1.035	[1.023, 1.047]	6.00***
NJ	0.008 (0.005)	1.008	[1.000, 1.017]	1.87
NM	0.041 (0.004)	1.041	[1.034, 1.049]	10.57***
NV	0.020 (0.005)	1.020	[1.011, 1.029]	4.24***
OH	-0.043 (0.003)	0.958	[0.952, 0.964]	-13.38***
OK	0.117 (0.003)	1.124	[1.117, 1.131]	37.61***
OR	-0.031 (0.004)	0.969	[0.962, 0.976]	-8.51***
PA	-0.023 (0.003)	0.978	[0.971, 0.984]	-7.13***
RI	0.246 (0.007)	1.278	[1.261, 1.296]	34.90***
SC	0.059 (0.004)	1.061	[1.053, 1.070]	14.41***
SD	0.083 (0.004)	1.086	[1.078, 1.095]	19.94***
TN	0.167 (0.003)	1.182	[1.175, 1.189]	53.58***
TX	0.126 (0.003)	1.135	[1.128, 1.142]	38.50***
UT	-0.295 (0.005)	0.745	[0.737, 0.752]	-56.06***
VA	0.091 (0.003)	1.096	[1.089, 1.102]	30.94***
VT	-0.020 (0.005)	0.980	[0.970, 0.990]	-3.83***
WA	0.033 (0.004)	1.033	[1.026, 1.041]	8.97***
WI	0.117 (0.003)	1.125	[1.117, 1.132]	35.25***
WV	0.060 (0.004)	1.062	[1.055, 1.069]	17.10***
WY	0.156 (0.004)	1.169	[1.159, 1.178]	38.47***

Notes: New York (NY) is the reference state. Significance levels: *** $p < 0.001$, ** $p < 0.01$, * $p < 0.05$. Model uses Gaussian GLM with log link function. Dependent variable is square root of PBL.

Table 16: State Fixed Effects for GLM-2a (Reference State: NY)

Variable	Coef. (Std. Error)	exp(Coef.)	95% CI	z-value
Lookahead (ref: 7-day)				
14-day ahead	0.113 (0.001)	1.119	[1.118, 1.121]	144.19***
21-day ahead	0.191 (0.001)	1.211	[1.209, 1.213]	250.41***
28-day ahead	0.262 (0.001)	1.300	[1.298, 1.302]	354.09***
Model Type (ref: Compartmental)				
Baseline Models	0.114 (0.002)	1.121	[1.118, 1.125]	73.38***
Deep Learning	0.037 (0.001)	1.037	[1.036, 1.039]	40.48***
Ensemble	0.007 (0.001)	1.007	[1.006, 1.009]	8.39***
Statistical	0.073 (0.001)	1.076	[1.074, 1.078]	90.26***
Mobility Used (ref: No)				
Not Applicable	-0.147 (0.001)	0.863	[0.861, 0.865]	-125.11***
Yes	-0.004 (0.001)	0.996	[0.994, 0.997]	-5.51***
Phase (ref: Phase 0)				
Phase 1	0.379 (0.003)	1.461	[1.453, 1.470]	130.08***
Phase 2	0.014 (0.003)	1.014	[1.008, 1.021]	4.25***
Phase 3	0.245 (0.003)	1.278	[1.270, 1.286]	76.61***
Phase 4	0.522 (0.003)	1.686	[1.676, 1.696]	170.89***
Phase 5	0.133 (0.004)	1.143	[1.134, 1.151]	35.21***
Phase 6	0.144 (0.004)	1.154	[1.146, 1.163]	37.40***
Health Outcomes				
Asthma	-0.045 (0.001)	0.956	[0.953, 0.958]	-35.31***
Obesity	0.004 (0.0001)	1.004	[1.004, 1.004]	31.23***
High Blood Pressure	-0.002 (0.0003)	0.998	[0.997, 0.998]	-7.78***
COPD	0.0002 (0.001)	1.000	[0.998, 1.002]	0.18
Stroke	-0.019 (0.003)	0.982	[0.975, 0.988]	-5.43***
Cancer	-0.016 (0.003)	0.984	[0.979, 0.990]	-5.60***
Coronary Heart Disease	0.084 (0.003)	1.088	[1.083, 1.093]	33.06***
Diabetes	-0.005 (0.001)	0.995	[0.993, 0.997]	-5.10***
Kidney Disease	0.043 (0.006)	1.044	[1.031, 1.057]	6.66***
Age 65+	0.102 (0.007)	1.107	[1.093, 1.122]	14.92***
Intercept	-4.746 (0.018)	0.009	[0.008, 0.009]	-263.71***

Notes: Urbanicity main effects and Urbanicity × Phase interactions shown in main manuscript table. Significance levels: *** $p < 0.001$, ** $p < 0.01$, * $p < 0.05$. Model uses Gaussian GLM with log link function. Dependent variable is square root of PBL.

Table 17: Main Effects for GLM-2b (Urbanicity × Phase Interactions)

State	Coef. (Std. Error)	exp(Coef.)	95% CI	z-value
AK	0.176 (0.005)	1.193	[1.182, 1.204]	38.81***
AL	0.073 (0.004)	1.075	[1.068, 1.083]	20.37***
AR	0.028 (0.004)	1.028	[1.020, 1.036]	7.05***
AZ	0.091 (0.005)	1.096	[1.085, 1.107]	18.27***
CA	0.021 (0.004)	1.022	[1.014, 1.029]	5.86***
CO	0.219 (0.004)	1.245	[1.236, 1.254]	62.14***
DE	0.040 (0.009)	1.041	[1.023, 1.060]	4.41***
GA	0.061 (0.004)	1.063	[1.055, 1.070]	17.12***
HI	-0.207 (0.011)	0.813	[0.796, 0.830]	-19.45***
IA	0.104 (0.004)	1.109	[1.101, 1.117]	28.83***
ID	0.088 (0.003)	1.092	[1.085, 1.099]	25.75***
IL	0.146 (0.003)	1.157	[1.150, 1.164]	47.03***
IN	0.082 (0.003)	1.085	[1.079, 1.092]	27.59***
KS	0.181 (0.003)	1.198	[1.191, 1.205]	59.89***
KY	0.146 (0.003)	1.157	[1.150, 1.164]	49.95***
LA	0.078 (0.004)	1.081	[1.072, 1.089]	19.99***
MA	-0.030 (0.005)	0.970	[0.960, 0.981]	-5.55***
MD	-0.069 (0.005)	0.933	[0.925, 0.942]	-15.25***
ME	-0.083 (0.005)	0.920	[0.911, 0.930]	-16.40***
MI	0.068 (0.003)	1.070	[1.064, 1.076]	22.85***
MN	0.113 (0.003)	1.119	[1.112, 1.127]	32.80***
MO	0.059 (0.003)	1.061	[1.054, 1.068]	17.34***
MS	0.074 (0.004)	1.077	[1.069, 1.086]	18.31***
MT	0.169 (0.003)	1.185	[1.177, 1.192]	50.20***
NC	0.028 (0.003)	1.028	[1.021, 1.035]	8.04***
ND	0.140 (0.004)	1.150	[1.141, 1.160]	33.30***
NE	0.068 (0.004)	1.070	[1.062, 1.079]	17.03***
NH	0.033 (0.006)	1.033	[1.022, 1.045]	5.76***
NJ	0.013 (0.004)	1.013	[1.004, 1.022]	2.92**
NM	0.037 (0.004)	1.038	[1.030, 1.046]	9.65***
NV	0.017 (0.005)	1.017	[1.007, 1.026]	3.55***
OH	-0.045 (0.003)	0.956	[0.950, 0.962]	-14.15***
OK	0.115 (0.003)	1.122	[1.115, 1.129]	37.16***
OR	-0.034 (0.004)	0.967	[0.960, 0.974]	-9.08***
PA	-0.024 (0.003)	0.976	[0.970, 0.982]	-7.68***
RI	0.252 (0.007)	1.287	[1.270, 1.305]	36.23***
SC	0.056 (0.004)	1.058	[1.049, 1.066]	13.66***
SD	0.080 (0.004)	1.084	[1.075, 1.093]	19.37***
TN	0.164 (0.003)	1.178	[1.171, 1.185]	52.72***
TX	0.123 (0.003)	1.131	[1.123, 1.138]	37.46***
UT	-0.299 (0.005)	0.742	[0.734, 0.749]	-56.89***
VA	0.089 (0.003)	1.093	[1.087, 1.100]	30.33***
VT	-0.025 (0.005)	0.975	[0.965, 0.985]	-4.75***
WA	0.031 (0.004)	1.031	[1.024, 1.039]	8.45***
WI	0.115 (0.003)	1.122	[1.115, 1.129]	34.65***
WV	0.058 (0.003)	1.060	[1.053, 1.067]	16.67***
WY	0.153 (0.004)	1.166	[1.156, 1.175]	37.90***

Notes: New York (NY) is the reference state. Significance levels: *** $p < 0.001$, ** $p < 0.01$, * $p < 0.05$. Model uses Gaussian GLM with log link function. Dependent variable is square root of PBL.

Table 18: State Fixed Effects for GLM-2b (Reference State: NY)

Variable	Coef. (Std. Error)	exp(Coef.)	95% CI	z-value
Lookahead (ref: 7-day)				
14-day ahead	0.113 (0.001)	1.119	[1.117, 1.121]	143.69***
21-day ahead	0.191 (0.001)	1.211	[1.209, 1.213]	249.56***
28-day ahead	0.262 (0.001)	1.299	[1.298, 1.301]	352.89***
Model Type (ref: Compartmental)				
Baseline Models	0.125 (0.004)	1.133	[1.125, 1.141]	34.04***
Deep Learning	0.055 (0.003)	1.056	[1.051, 1.062]	20.41***
Ensemble	0.017 (0.002)	1.017	[1.013, 1.021]	8.45***
Statistical	0.050 (0.002)	1.051	[1.046, 1.056]	22.23***
Mobility Used (ref: No)				
Not Applicable	-0.147 (0.001)	0.863	[0.861, 0.865]	-124.79***
Yes	-0.005 (0.001)	0.995	[0.994, 0.997]	-6.21***
Phase (ref: Phase 0)				
Phase 1	0.368 (0.001)	1.445	[1.442, 1.448]	377.50***
Phase 2	-0.117 (0.001)	0.890	[0.888, 0.892]	-100.13***
Phase 3	0.250 (0.001)	1.284	[1.282, 1.287]	236.13***
Phase 4	0.439 (0.001)	1.552	[1.549, 1.555]	416.75***
Phase 5	-0.031 (0.001)	0.969	[0.967, 0.972]	-22.75***
Phase 6	0.141 (0.001)	1.151	[1.148, 1.154]	112.27***
Health Outcomes				
Asthma	-0.045 (0.001)	0.956	[0.954, 0.958]	-34.84***
Obesity	0.004 (0.0001)	1.004	[1.004, 1.005]	31.87***
High Blood Pressure	-0.002 (0.0003)	0.998	[0.997, 0.998]	-7.68***
COPD	-0.0001 (0.001)	1.000	[0.998, 1.002]	-0.06
Stroke	-0.019 (0.003)	0.981	[0.974, 0.988]	-5.60***
Cancer	-0.016 (0.003)	0.984	[0.978, 0.989]	-5.69***
Coronary Heart Disease	0.085 (0.003)	1.089	[1.084, 1.095]	33.34***
Diabetes	-0.005 (0.001)	0.995	[0.993, 0.997]	-5.41***
Kidney Disease	0.042 (0.006)	1.043	[1.030, 1.056]	6.59***
Age 65+	0.105 (0.007)	1.110	[1.096, 1.125]	15.29***
Intercept	-4.710 (0.018)	0.009	[0.009, 0.009]	-262.75***

Notes: Urbanicity main effects and Urbanicity × Model Type interactions shown in main manuscript table. Significance levels: *** $p < 0.001$, ** $p < 0.01$, * $p < 0.05$. Model uses Gaussian GLM with log link function. Dependent variable is square root of PBL.

Table 19: Main Effects for GLM-2c (Urbanicity × Model Type Interactions)

State	Coef. (Std. Error)	exp(Coef.)	95% CI	z-value
AK	0.181 (0.005)	1.198	[1.188, 1.209]	39.68***
AL	0.076 (0.004)	1.079	[1.071, 1.086]	21.12***
AR	0.030 (0.004)	1.030	[1.022, 1.038]	7.50***
AZ	0.095 (0.005)	1.099	[1.088, 1.110]	18.85***
CA	0.026 (0.004)	1.026	[1.019, 1.034]	7.08***
CO	0.222 (0.004)	1.249	[1.240, 1.257]	62.76***
DE	0.039 (0.009)	1.040	[1.021, 1.059]	4.26***
GA	0.064 (0.004)	1.066	[1.059, 1.074]	18.04***
HI	-0.204 (0.011)	0.816	[0.799, 0.833]	-19.08***
IA	0.106 (0.004)	1.112	[1.104, 1.120]	29.46***
ID	0.090 (0.003)	1.095	[1.087, 1.102]	26.34***
IL	0.148 (0.003)	1.160	[1.153, 1.167]	47.60***
IN	0.085 (0.003)	1.088	[1.082, 1.095]	28.43***
KS	0.183 (0.003)	1.200	[1.193, 1.207]	60.22***
KY	0.148 (0.003)	1.160	[1.153, 1.166]	50.58***
LA	0.081 (0.004)	1.084	[1.076, 1.092]	20.70***
MA	-0.032 (0.005)	0.969	[0.959, 0.979]	-5.78***
MD	-0.069 (0.005)	0.933	[0.925, 0.941]	-15.21***
ME	-0.080 (0.005)	0.923	[0.914, 0.933]	-15.73***
MI	0.070 (0.003)	1.072	[1.066, 1.079]	23.39***
MN	0.115 (0.003)	1.122	[1.114, 1.129]	33.30***
MO	0.062 (0.003)	1.064	[1.057, 1.071]	18.01***
MS	0.077 (0.004)	1.080	[1.072, 1.089]	18.90***
MT	0.171 (0.003)	1.187	[1.179, 1.195]	50.62***
NC	0.031 (0.003)	1.031	[1.024, 1.038]	8.84***
ND	0.142 (0.004)	1.153	[1.143, 1.162]	33.67***
NE	0.071 (0.004)	1.074	[1.065, 1.082]	17.79***
NH	0.034 (0.006)	1.035	[1.023, 1.047]	5.99***
NJ	0.009 (0.005)	1.009	[1.000, 1.018]	1.91
NM	0.040 (0.004)	1.041	[1.033, 1.049]	10.50***
NV	0.020 (0.005)	1.020	[1.010, 1.029]	4.19***
OH	-0.043 (0.003)	0.958	[0.952, 0.964]	-13.38***
OK	0.117 (0.003)	1.124	[1.117, 1.131]	37.54***
OR	-0.032 (0.004)	0.969	[0.962, 0.976]	-8.55***
PA	-0.023 (0.003)	0.977	[0.971, 0.984]	-7.16***
RI	0.246 (0.007)	1.279	[1.261, 1.296]	34.90***
SC	0.059 (0.004)	1.061	[1.053, 1.070]	14.38***
SD	0.083 (0.004)	1.086	[1.077, 1.095]	19.86***
TN	0.167 (0.003)	1.182	[1.175, 1.189]	53.55***
TX	0.126 (0.003)	1.135	[1.127, 1.142]	38.44***
UT	-0.295 (0.005)	0.745	[0.737, 0.752]	-56.01***
VA	0.091 (0.003)	1.096	[1.089, 1.102]	30.91***
VT	-0.020 (0.005)	0.980	[0.970, 0.990]	-3.86***
WA	0.033 (0.004)	1.033	[1.026, 1.041]	8.97***
WI	0.117 (0.003)	1.124	[1.117, 1.132]	35.16***
WV	0.060 (0.004)	1.062	[1.055, 1.069]	17.09***
WY	0.156 (0.004)	1.169	[1.159, 1.178]	38.38***

Notes: New York (NY) is the reference state. Significance levels: *** $p < 0.001$, ** $p < 0.01$, * $p < 0.05$. Model uses Gaussian GLM with log link function. Dependent variable is square root of PBL.

Table 20: State Fixed Effects for GLM-2c (Reference State: NY)

**Variable**	**Coef. (Std. Error)**	**exp(Coef.)**	**95% CI**	**z-value**
Lookahead (ref: 7-day)				
14-day ahead	0.113 (0.001)	1.119	[1.117, 1.121]	143.72***
21-day ahead	0.191 (0.001)	1.211	[1.209, 1.213]	249.59***
28-day ahead	0.262 (0.001)	1.300	[1.298, 1.301]	352.91***
Model Type (ref: Compartmental)				
Baseline Models	0.114 (0.002)	1.121	[1.118, 1.124]	73.05***
Deep Learning	0.037 (0.001)	1.037	[1.035, 1.039]	40.28***
Ensemble	0.007 (0.001)	1.007	[1.005, 1.009]	7.94***
Statistical	0.073 (0.001)	1.076	[1.075, 1.078]	90.18***
Mobility Used (ref: No)				
Not Applicable	-0.127 (0.003)	0.881	[0.876, 0.885]	-49.05***
Yes	0.023 (0.002)	1.023	[1.019, 1.027]	11.60***
Phase (ref: Phase 0)				
Phase 1	0.368 (0.001)	1.445	[1.442, 1.448]	377.52***
Phase 2	-0.117 (0.001)	0.890	[0.888, 0.892]	-100.12***
Phase 3	0.250 (0.001)	1.284	[1.282, 1.287]	236.12***
Phase 4	0.440 (0.001)	1.552	[1.549, 1.555]	416.76***
Phase 5	-0.031 (0.001)	0.969	[0.967, 0.972]	-22.80***
Phase 6	0.141 (0.001)	1.151	[1.148, 1.154]	112.26***
Health Outcomes				
Asthma	-0.045 (0.001)	0.956	[0.954, 0.958]	-34.87***
Obesity	0.004 (0.0001)	1.004	[1.004, 1.005]	31.88***
High Blood Pressure	-0.002 (0.0003)	0.998	[0.997, 0.998]	-7.66***
COPD	-0.0001 (0.001)	1.000	[0.998, 1.002]	-0.06
Stroke	-0.019 (0.003)	0.981	[0.975, 0.988]	-5.56***
Cancer	-0.016 (0.003)	0.984	[0.978, 0.989]	-5.72***
Coronary Heart Disease	0.085 (0.003)	1.089	[1.084, 1.095]	33.37***
Diabetes	-0.005 (0.001)	0.995	[0.993, 0.997]	-5.45***
Kidney Disease	0.042 (0.006)	1.043	[1.030, 1.056]	6.58***
Age 65+	0.105 (0.007)	1.111	[1.096, 1.126]	15.31***
Intercept	-4.729 (0.018)	0.009	[0.009, 0.009]	-263.34***

Notes: Urbanicity main effects and Urbanicity × Mobility interactions shown in main manuscript table. Significance levels: *** $p < 0.001$, ** $p < 0.01$, * $p < 0.05$. Model uses Gaussian GLM with log link function. Dependent variable is square root of PBL.

Table 21: Main Effects for GLM-2d (Urbanicity × Mobility Interactions)

State	Coef. (Std. Error)	exp(Coef.)	95% CI	z-value
AK	0.181 (0.005)	1.198	[1.187, 1.209]	39.65***
AL	0.076 (0.004)	1.078	[1.071, 1.086]	21.11***
AR	0.029 (0.004)	1.030	[1.022, 1.038]	7.46***
AZ	0.094 (0.005)	1.099	[1.088, 1.110]	18.82***
CA	0.026 (0.004)	1.026	[1.019, 1.034]	7.06***
CO	0.222 (0.004)	1.249	[1.240, 1.257]	62.72***
DE	0.039 (0.009)	1.040	[1.021, 1.059]	4.23***
GA	0.064 (0.004)	1.066	[1.059, 1.074]	18.03***
HI	-0.204 (0.011)	0.815	[0.798, 0.833]	-19.10***
IA	0.106 (0.004)	1.112	[1.104, 1.120]	29.44***
ID	0.091 (0.003)	1.095	[1.087, 1.102]	26.35***
IL	0.148 (0.003)	1.160	[1.153, 1.167]	47.58***
IN	0.085 (0.003)	1.088	[1.082, 1.095]	28.42***
KS	0.183 (0.003)	1.200	[1.193, 1.207]	60.21***
KY	0.148 (0.003)	1.160	[1.153, 1.166]	50.58***
LA	0.081 (0.004)	1.084	[1.076, 1.092]	20.69***
MA	-0.032 (0.005)	0.969	[0.959, 0.979]	-5.77***
MD	-0.069 (0.005)	0.933	[0.925, 0.941]	-15.16***
ME	-0.080 (0.005)	0.923	[0.914, 0.932]	-15.74***
MI	0.070 (0.003)	1.072	[1.066, 1.078]	23.37***
MN	0.115 (0.003)	1.122	[1.114, 1.129]	33.29***
MO	0.062 (0.003)	1.064	[1.057, 1.071]	18.00***
MS	0.077 (0.004)	1.080	[1.071, 1.089]	18.88***
MT	0.171 (0.003)	1.187	[1.179, 1.195]	50.62***
NC	0.031 (0.003)	1.031	[1.024, 1.038]	8.81***
ND	0.142 (0.004)	1.153	[1.143, 1.162]	33.66***
NE	0.071 (0.004)	1.074	[1.065, 1.082]	17.75***
NH	0.034 (0.006)	1.035	[1.023, 1.047]	6.01***
NJ	0.009 (0.005)	1.009	[1.000, 1.018]	1.92
NM	0.040 (0.004)	1.041	[1.033, 1.049]	10.50***
NV	0.020 (0.005)	1.020	[1.010, 1.029]	4.17***
OH	-0.043 (0.003)	0.958	[0.952, 0.964]	-13.40***
OK	0.117 (0.003)	1.124	[1.117, 1.131]	37.53***
OR	-0.032 (0.004)	0.969	[0.962, 0.976]	-8.55***
PA	-0.023 (0.003)	0.977	[0.971, 0.983]	-7.20***
RI	0.246 (0.007)	1.278	[1.261, 1.296]	34.87***
SC	0.059 (0.004)	1.061	[1.052, 1.070]	14.36***
SD	0.083 (0.004)	1.086	[1.077, 1.095]	19.83***
TN	0.167 (0.003)	1.182	[1.175, 1.189]	53.52***
TX	0.126 (0.003)	1.135	[1.127, 1.142]	38.42***
UT	-0.295 (0.005)	0.744	[0.737, 0.752]	-56.02***
VA	0.091 (0.003)	1.096	[1.089, 1.102]	30.92***
VT	-0.020 (0.005)	0.980	[0.970, 0.990]	-3.87***
WA	0.033 (0.004)	1.033	[1.026, 1.041]	8.97***
WI	0.117 (0.003)	1.124	[1.117, 1.132]	35.15***
WV	0.060 (0.004)	1.062	[1.055, 1.069]	17.09***
WY	0.156 (0.004)	1.169	[1.159, 1.178]	38.38***

Notes: New York (NY) is the reference state. Significance levels: *** $p < 0.001$, ** $p < 0.01$, * $p < 0.05$. Model uses Gaussian GLM with log link function. Dependent variable is square root of PBL.

Table 22: State Fixed Effects for GLM-2d (Reference State: NY)

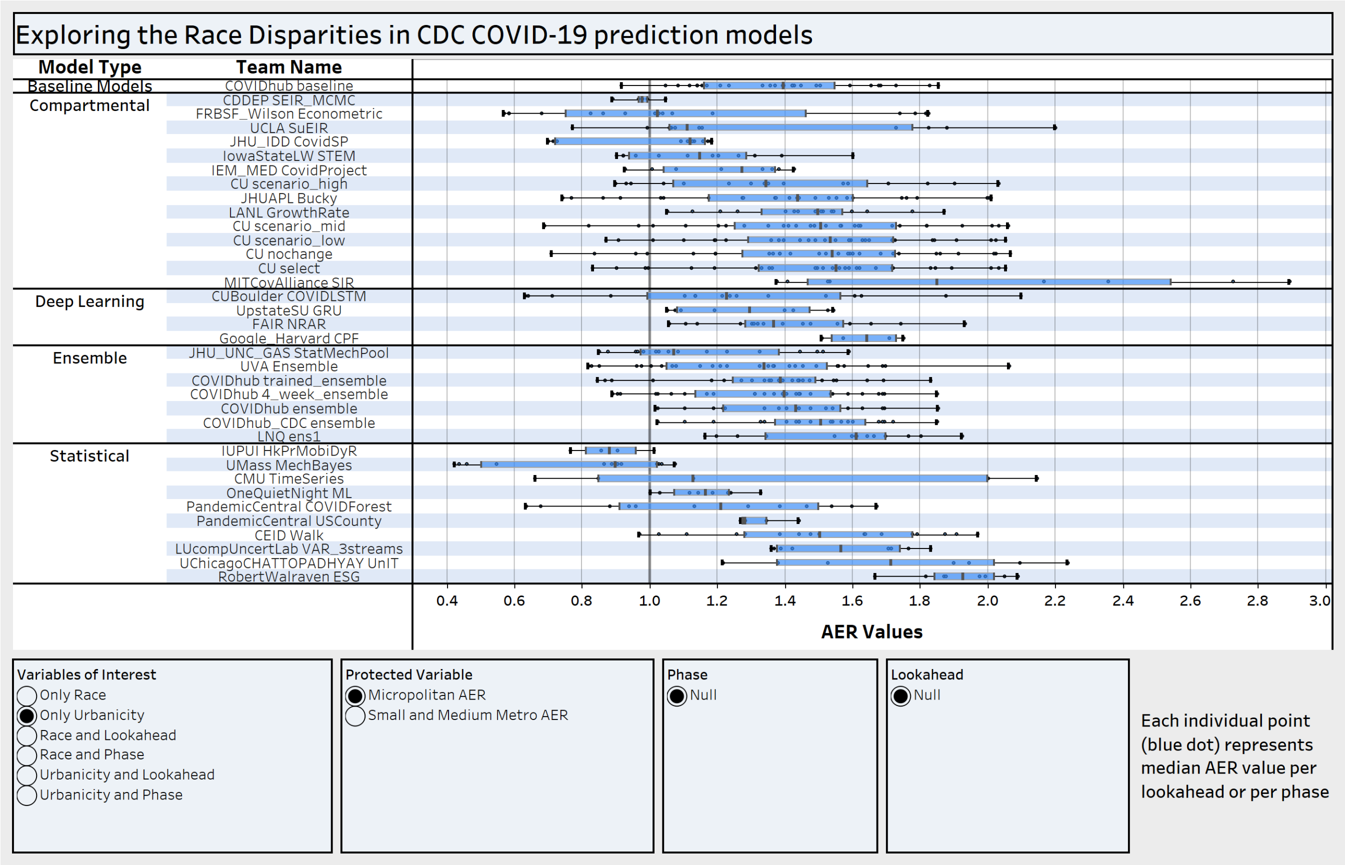

Figure 7: Forecast Hub Fairness Dashboard showing AER distribution for urbanization levels. Models are organized by type, with teams sorted within each type by median AER values. The AER values compare prediction errors between Micropolitan areas and Large Metropolitan areas (reference group), where values above 1.0 indicate higher prediction errors for Micropolitan regions.

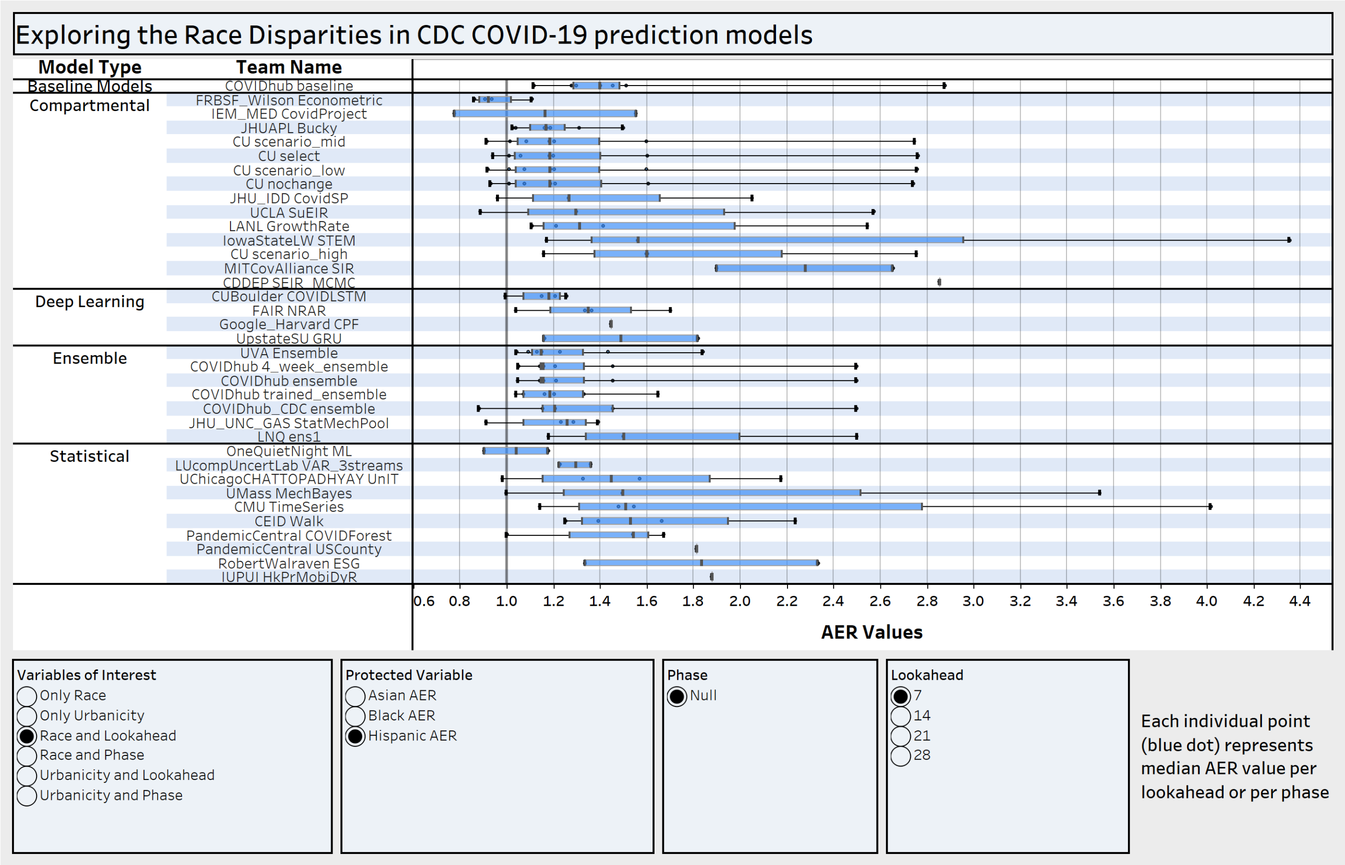

Figure 8: Forecast Hub Fairness Dashboard displaying the interaction between race and forecast horizon (lookahead). The visualization shows how Hispanic-White AER values vary across different forecast periods (7, 14, 21, and 28 days), allowing assessment of how prediction fairness changes with forecast horizon length(lookaheads).

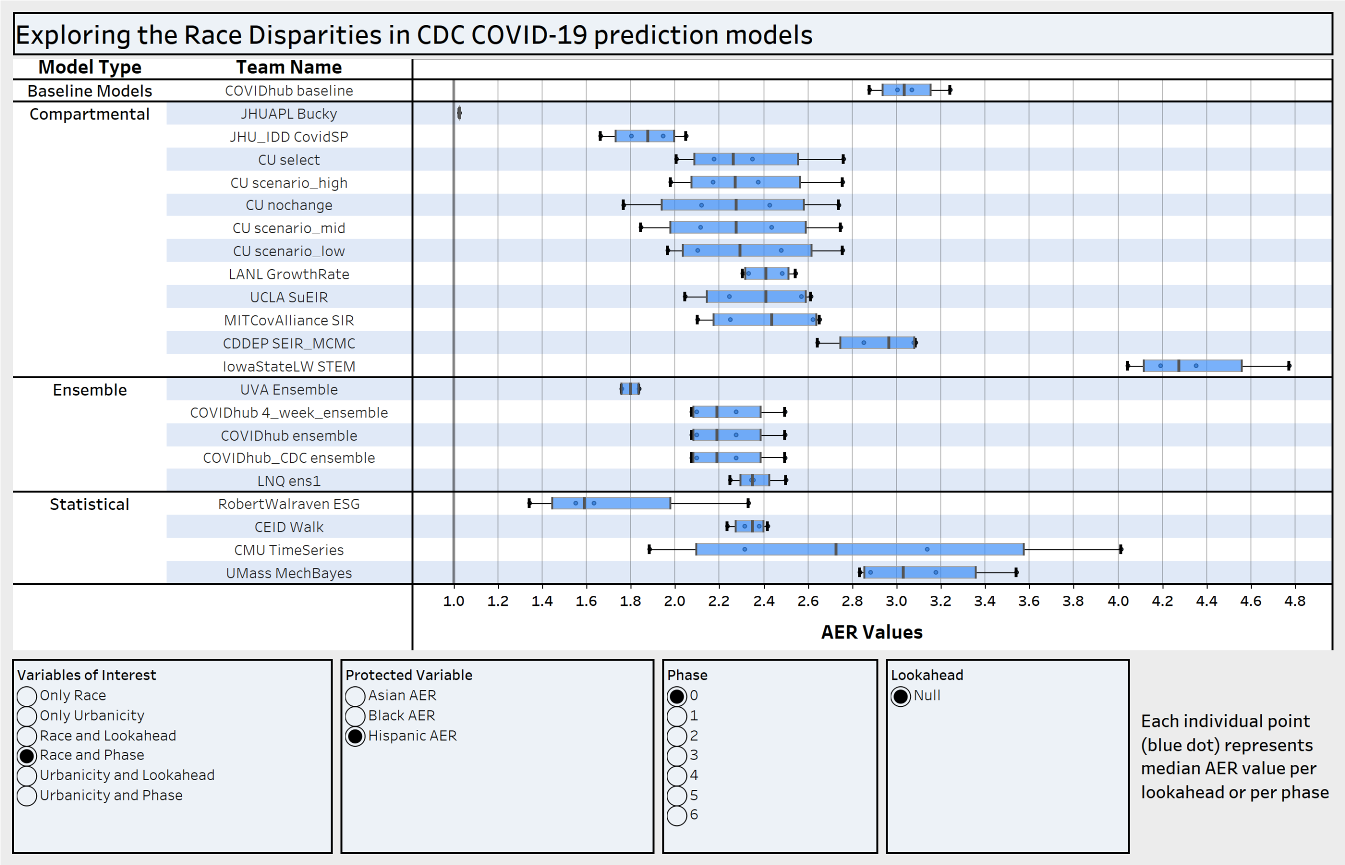

Figure 9: Forecast Hub Fairness Dashboard illustrating the relationship between race and pandemic phases. The visualization reveals how Hispanic-White AER values evolved across different pandemic phases (0-6), enabling analysis of how prediction fairness varied during different stages of the pandemic.

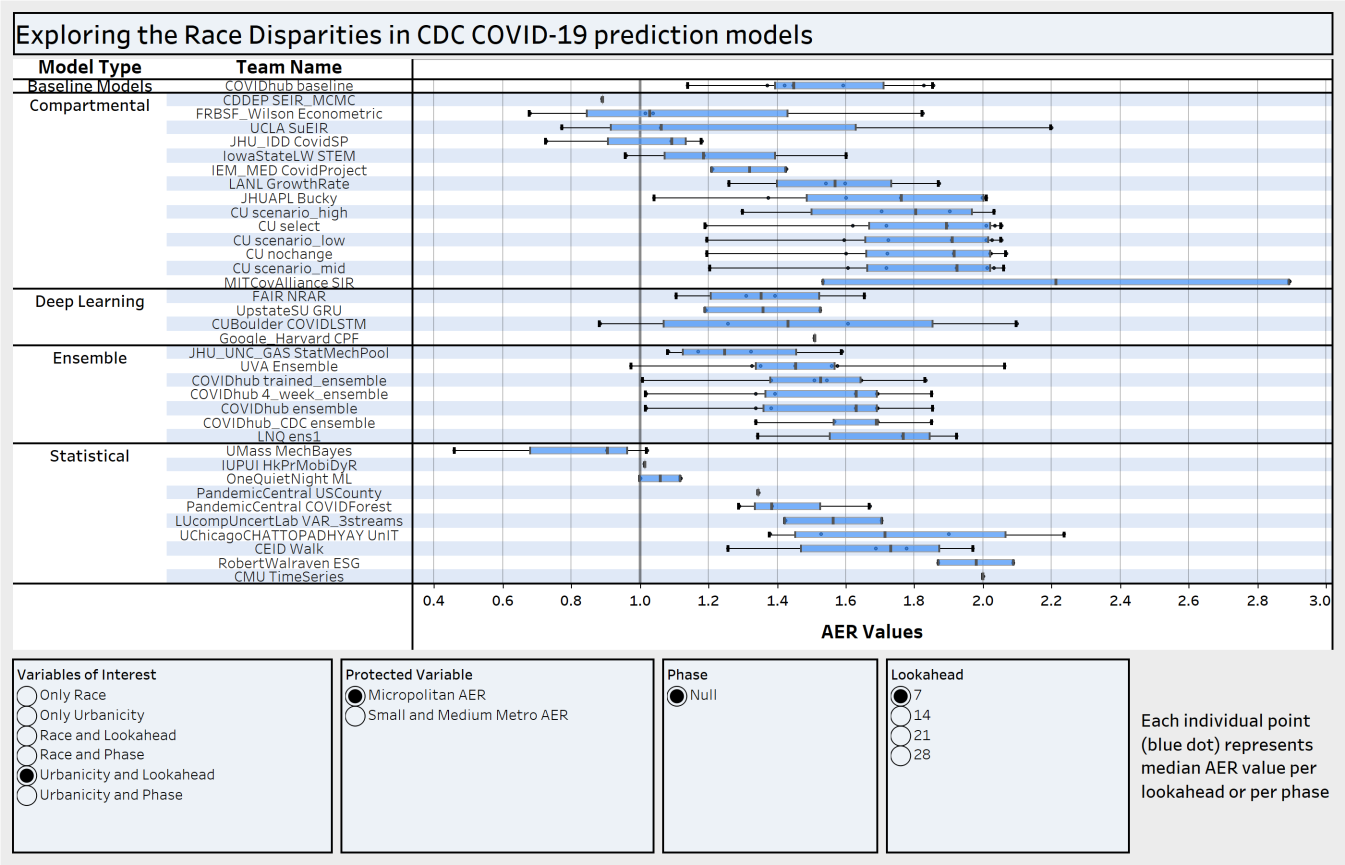

Figure 10: Forecast Hub Fairness Dashboard showing the interaction between urbanization levels and forecast horizon. The visualization demonstrates how Micropolitan-Large Metropolitan AER values change across different forecast periods (7, 14, 21, and 28 days), highlighting the relationship between prediction fairness and forecast horizon lengths (lookaheads).

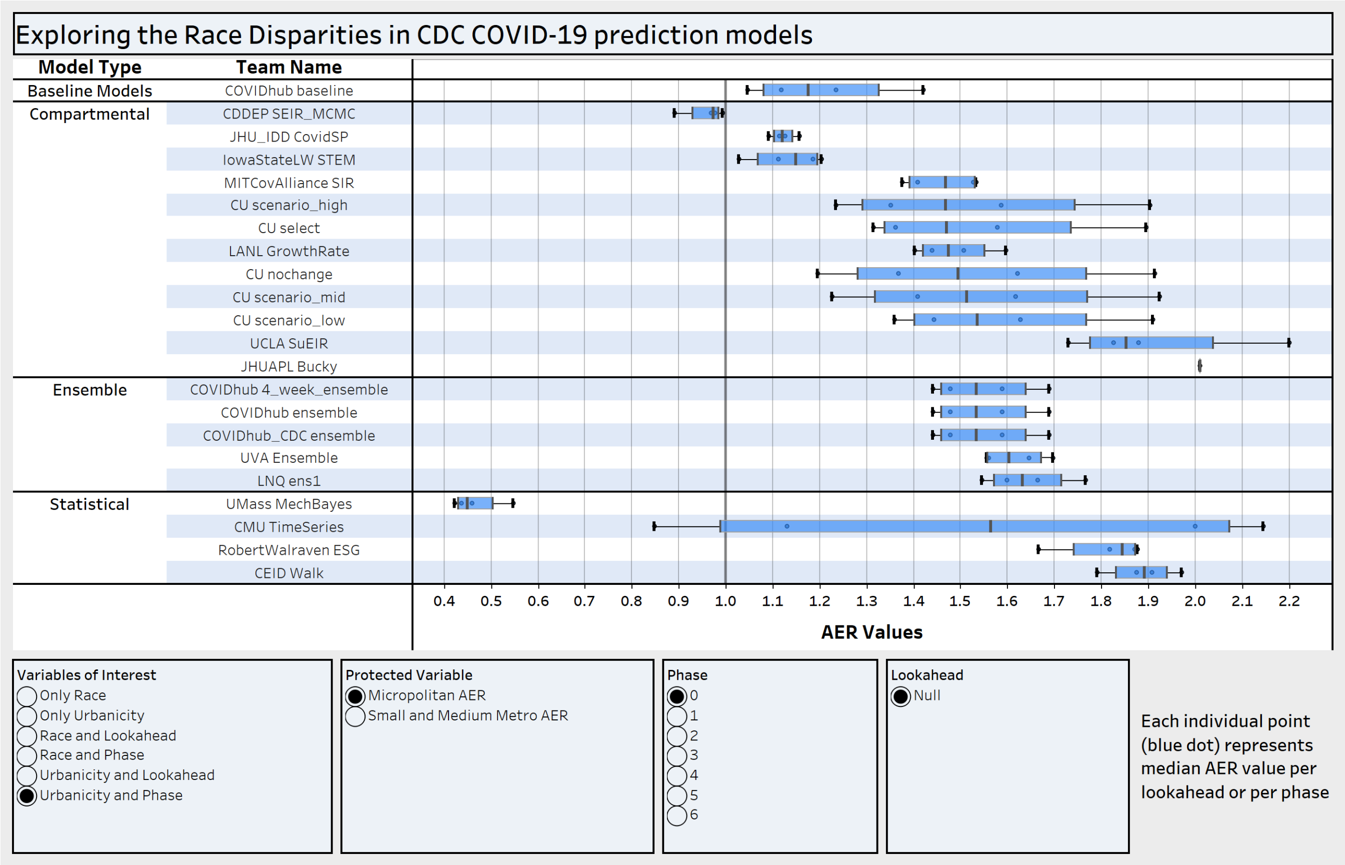

Figure 11: Forecast Hub Fairness Dashboard depicting the relationship between urbanization levels and pandemic phases. The visualization shows how Micropolitan-Large Metropolitan AER values varied across different pandemic phases (0-6), revealing temporal patterns in prediction fairness across the urban-rural spectrum.
