# Supplementary material for: Auditing the fairness of the US COVID-19 forecast hub’s case prediction models
Source: PLoS One. 2025 Apr 22;20(4):e0319383. doi: 10.1371/journal.pone.0319383 (PMC12014149; doi:10.1371/journal.pone.0319383)
Supplement: Attachment — Submitted filename: Review PLOS ONE (CDC FORECASTHUB).pdf [file pone.0319383.s002.pdf]

**Reviewer #1:**

We thank the reviewer for the critical feedback provided. We have responded to each individual comment in this document, and we have updated the main manuscript and the appendix accordingly.

The statistics seem sound and the justification of the study is clear but I have questions and comments about the fairness card and dashboard.

The manuscript should include a data availability statement. I think it is reasonable not to provide access to the dashboard before publication but it more explanation is required for the review.

The datasets used in this article are publicly available in the following repositories:

1. All COVID-19 Forecasts are available at:
https://github.com/reichlab/covid19-forecast-hub/tree/master

2. All COVID-19 Ground Truth Data are available at:
https://github.com/CSSEGISandData/COVID-19

3. All Race and Ethnicity, Population Data are available at:
https://www.census.gov/data/tables/time-series/demo/popest/2020s-counties-detail.html

4. The Urban-Rural Classification Scheme for Counties:
https://www.cdc.gov/nchs/data_access/urban_rural.htm\#2013_Urban-Rural_Classification_Scheme_for_Counties

5. All Health Outcome Datasets are provided at:
https://data.cdc.gov/500-Cities-Places/PLACES-Local-Data-for-Better-Health-Place-Data-202/eav7-hnsx/about_data

All datasets used are publicly available. We have added the data availability statement in the submission portal, and all links are also available in the paper as references.

Fairness nutritional card – in what way is this study about food?

We have clarified this in the new version of the paper (section Dashboard). 'Nutritional labels' were proposed by Stoyanovich and Howe to assess model fairness [1] drawing an analogy to the food industry, where simple, standard labels convey information about the ingredients and production processes. We have adapted these cards to the COVID-19 fairness context, as a way to provide detailed COVID-19 forecast model fairness information.

[1] Stoyanovich J, Howe B. Nutritional labels for data and models. A Quarterly bulletin of the Computer Society of the IEEE Technical Committee on Data Engineering. 2019;42(3)

Inconsistency of “COVID-19” and “covid-19”.

We have addressed and removed the inconsistencies.

The dashboard should be self-explanatory and easy to read.
(i) The meanings of the dots, blue shading, horizontal lines between dots (and no horizontal lines between dots) for the AERs should be explained

We have extensively edited the Dashboard section to clarify its meaning and use. In addition, we have clearly explained the meaning of the different elements raised by the reviewer in the visualization in Figure 3 (legend). Specifically:

1. For each individual model, we compute the median AER for each lookahead and each phase. Each individual dot represents the median AER value per lookahead or per phase.
2. The blue shading shows the interquartile range (IQR), containing 50% of the model's AER values
3. The central vertical line within the blue shading indicates the overall median AER across all phases and lookaheads.
4. Thin vertical lines (whiskers) extend to show the minimum and maximum AER values, excluding statistical outliers.

(ii) It is not clear what the plot would look like with Race and Lookahead or Race with phase (reporting more than one view of the dashboard would be useful).

We have added a description of the different types of Dashboard views available in the main paper (“Dashboard” section). In addition, we have added plots for each visualization type in the S1 Appendix (with links to them in the main paper, see Figures 7-11).

(iii) Adding the dates of data would be useful to give context to the data.

Predictions in the dashboard can be explored by phase, with phases being defined by COVID-19 case volume patterns as described in section “Model-Data Characteristics” in page 6. The phases themselves capture the meaningful temporal patterns in the pandemic's progression - from periods of low case volumes to peak waves - which is more relevant for understanding how model fairness varied across different epidemiological contexts than calendar dates.

(iv) It would be helpful to give a simple example of how the dashboard would be used (and therefore demonstrate its usefulness) e.g. interpreting IowaStateLW STEM (wide range of AERs) vs LUcompUncertLab Var_3streams (narrow and lower range of AERs) for “Only Race” and “Hispanic AER” variable i.e. the latter model scores higher on fairness?

To illustrate the dashboard's utility in assessing model fairness, we have extensively edited the Dashboard section with a subsection called “Sample Use Case” (page 24). In this sample use case, we explore how the dashboard and the nutritional cards (Figures 3 and 4) can be used to make decisions about the fairness of the models.

For the specific example raised by the reviewer, we have added the following text to the main paper: “The IowaStateLW STEM model exhibits a wide range of median AER values (min: 1.172, max: 4.772, median: 1.588) across its predictions, indicating highly variable fairness performance when assessed across different phases and lookaheads. On the other hand, the LUcompUncertLab VAR_3streams model shows consistent median AER values within a small range from 1.228 to 1.334 (median: 1.280). Comparing both models, we observe that although both of them are systematically producing predictions that are less fair for Hispanic counties (AER values are larger than 1), the LUcompUncertLab VAR_3streams model has lower max AER values, suggesting that model might be a better choice.”

It is important to note that while the box plots reveal the distribution of median AER values (calculated separately for each phase or lookahead period), direct comparisons between models should be made cautiously as they may have predictions for different numbers of phases (for example IowaStateLW STEM has predictions for Phase 0,1,2 and LUcompUncertLab VAR_3streams has predictions for Phase 5,6). Hence, we recommend doing this analysis in combination with Figure 1 in S1 Appendix. Overall, this type of analysis helps researchers and decision-makers understand individual model's fairness implications across different demographic groups, providing insights beyond traditional accuracy metrics.

(v) Why are the Phase and Lookahead options necessary as options for Variables of Interest seem to cover both ?

The Phase and Lookahead options serve a different purpose from the Variables of Interest selection. While Variables of Interest allows users to examine how AERs vary across all phases or lookaheads (showing the complete distribution), the individual Phase and Lookahead selectors enable users to filter the data to a specific phase or prediction horizon. This granular filtering capability allows researchers to investigate fairness metrics under particular temporal conditions - for example, examining model performance specifically during peak case periods (a particular phase) or for short-term predictions (a specific lookahead). For detailed examples of how these filtering options reveal different patterns in model fairness, we refer the reviewer to the newly edited Dashboard section (page 23) as well as to Figures 8-11 in S1 Appendix, which demonstrate how AER distributions can vary substantially when examined at specific temporal granularities.

(vi) Spaces between the model names would be helpful as it is currently difficult to match the AERs to the models

We have added row banding for clearer distinction between models.

(vii) It would be helpful to provide options to: (a) sort the models in a different way e.g by fairness based on the upper limit of the AER trace; (b) subset models e.g. all with *** (shading out the others); and (c) highlight individual models

We have sorted the models per model type in increasing order of median AER value.

**Reviewer #2:**

We thank the reviewer for the critical feedback provided. We have responded to each individual comment in this document, and we have updated the main manuscript and the appendix accordingly.

The authors present a critical study of several COVID-19 prediction models that utilize U.S. data from the Forecast Hub COVID-19 platform. They propose incorporating race, ethnicity, and rural-urban characteristics to assess the predictions of 36 models, categorized as ensemble, statistical, compartmental, deep learning, and baseline models. Their analysis shows that the models do not consistently perform well across different social determinants.
The study provides results based on various scenarios, including mobility data, lookaheads, and different COVID-19 phases. The analytical approach is clearly explained and appears reasonable.

I suggest addressing the following issue before publication:

First of all, I suggest to the authors soften some of the statements that come across as harsh regarding the performance of the various models. Many researchers developed these models quickly in order to provide insights into the dynamics of the rapidly spreading pandemic. Most of these researchers contributed to the development of predictive models, even while working on other research projects, due to the global urgency. Therefore, I recommend that the authors avoid a polemical tone in the article, considering the historical context in which the models were proposed. Instead, they should encourage more precise research in the future without undermining the efforts made by researchers during the spread of COVID-19. In any case, I believe that the efforts of numerous researchers around the world during this tragic period should be appreciated.

We agree with the reviewer, and we highly value the work that our colleagues have done in the development of complex COVID-19 forecast models. Hence, we have reframed some of our statements particularly in the introduction, presenting fairness evaluation as an opportunity for enhancement rather than a limitation. For example, we revised: "the Forecast Hub fails to evaluate..." to "While the Forecast Hub has made significant contributions through its accuracy-focused predictions..., there is an opportunity to expand its evaluation framework…

Throughout the manuscript, we have maintained our focus on constructively identifying opportunities for improvement while acknowledging the valuable work done under extremely challenging circumstances. We kindly ask the reviewer to check highlighted changes in the Introduction,

In addition:

1 Clarify the Focus on U.S. Data: The paper should explicitly state that the comparisons are based on U.S. data and models. There is also a European Forecast Hub producing similar predictions. However, in Europe, particularly in countries like Italy, race differences may be less pronounced due to the public healthcare system. It is not clearly specified in the paper that the analysis pertains to U.S. data and models.

We have made necessary changes to the title, abstract and main manuscript to specify we have focused our work on the US CDC ForecastHub.

2 Reduce Repetitions: Some sentences are repeated multiple times throughout the paper. After reading the entire paper, I suggest revising these repetitions for clarity and conciseness.

We have extensively edited the paper to reduce repetitions. We have also simplified our analytical approach with a focus on the regression analysis instead of the majority, plurality and regression methods, which added a lot of repetitions.

3 Ensure Replicability and possibly reproducibility: To make this research replicable, please confirm whether the data and code used for the analysis are publicly available.

All the datasets used in this study are publicly available, and we have specified that in our manuscript. The code will be made publicly available upon publication.

The datasets supporting the conclusions of this article are available in the following repositories:

1. All COVID-19 Forecasts are available at:
https://github.com/reichlab/covid19-forecast-hub/tree/master

2. All COVID-19 Ground Truth Data are available at:
https://github.com/CSSEGISandData/COVID-19

3. All Race and Ethnicity, Population Data are available at:
https://www.census.gov/data/tables/time-series/demo/popest/2020s-counties-detail.html

4. The Urban-Rural Classification Scheme for Counties:
https://www.cdc.gov/nchs/data_access/urban_rural.htm\#2013_Urban-Rural_Classification_Scheme_for_Counties

5. All Health Outcome Datasets are provided at: https://data.cdc.gov/500-Cities-Places/PLACES-Local-Data-for-Better-Health-Place-Data-202/eav7-hnsx/about_data

We have also added the data availability statement in the submission portal.

4 It would be more appropriate to cite each model presented in Figure 8 and in the Appendix with proper references to the authors. These citations should be available on the Forecast Hub website.

We have added links to each paper and model in the Acknowledgements section.

**Reviewer #3:**

We thank the reviewer for the critical feedback provided. We have responded to each individual comment in this document, and we have updated the main manuscript and the appendix accordingly.

We would like to highlight a major change in the analytical approach. As identified by the reviewers, the paper was a bit long and repetitive at times. This was mostly due to the three race association approaches we proposed: majority, plurality and regression. To simplify the paper, and to be able to provide an in-depth analysis of our modeling choices, we have focused only on the regression approach. We kindly ask the reviewer to check our narrowed down approach in the "Analytical Approach" section (pages 6-10).

The manuscript provides a valuable case study on applying fairness to evaluate COVID-19 models in the United States. This is an important contribution, addressing a significant gap in the current modeling literature. The work is commendable in its attempt to assess the performance of models. However, the paper is notably long for an epidemiological study, and the authors might consider condensing certain sections to enhance their message. Additionally, I offer the following observations for consideration:

1) In the abstract, the phrase "we show […] performance across social determinants, with minority racial and ethnic groups as well as less urbanized areas" suggests the inclusion of multiple social determinants. However, the manuscript primarily focuses on racial factors. Please clarify if other social determinants were indeed analyzed or revise the wording.

We have updated the paper to clarify that our focus is on two social determinants only: race/ethnicity and urbanization levels.

2) In line 54, the authors assert that self-reported race is the "most accurate source and prevents errors." While I agree, this warrants a citation.

We have added a relevant citation in line 54: https://pmc.ncbi.nlm.nih.gov/articles/PMC1447261/

3) The classification of model types requires further elaboration. The categories used are not immediately intuitive. For instance (as I understand it) the IEM_MED-CovidProject, incorporates deep learning into a compartmental model.

We have added an extensive discussion of our classification model approach in the S1 Appendix (Section 1.2). We kindly ask the reviewer to check the added explanations.

Regarding the IEM_MED-CovidProject model specifically, while it uses AI techniques for parameter fitting, we classify it as compartmental because its core predictive mechanism relies on a SEIR framework, with AI serving an auxiliary role in parameter optimization. This classification prioritizes the fundamental epidemiological structure over supplementary computational techniques used for implementation. This taxonomy acknowledges that modern epidemiological models often incorporate multiple computational techniques, but classifies them based on their primary predictive mechanism rather than auxiliary methods used for implementation or optimization. We recognize that some models may exhibit hybrid characteristics, but we assign them to categories based on their dominant modeling paradigm to enable meaningful comparative analysis.

4) The criteria used to delineate COVID-19 phases should be expanded upon. For example, Phases 5 and 6 appear to represent the same pandemic wave. A clearer justification for these phase distinctions is necessary.

We kindly ask the reviewer to check the elaborate discussion we have added on this in Appendix S1, Section 1.3. In short, we divided the pandemic timeline into distinct phases using a data-driven approach based on local minima (valleys) and local maxima (peaks) in COVID-19 case volumes. As for the distinction between P5 and P6, P6 represents a distinct final phase marked by a sustained plateau of ~130,000 cases that emerged after two clear valleys in the case curve, creating a natural demarcation point to evaluate model performance during periods of sustained transmission.

5) In the Plurality Approach, why were the Non-White used in the Majority dropped? Please elaborate. There seems to be an invisibilization of some minorities (such as Native Americans) that under this approach are converted to white (look at Alaska, for example). A paper on fairness should not exclude minorities without a careful explanation.

As mentioned earlier, we have removed the plurality and majority approaches from the paper for clarity purposes, using only the regression approach that accounts for the exact percentage distribution of each race/ethnicity per county. However, we agree with the reviewer in that a clearer explanation regarding our choices with respect to racial and ethnic groups is necessary.

For that purpose, we have added a comprehensive discussion and justification of our choices in Section 1.1 in the S1 Appendix. We have also added a brief description and a table (Table 1) in the main text.

To briefly answer the question raised by the reviewer, Non-Hispanic Native Hawaiian/Pacific Islander (NHPI) and Non-Hispanic American Indian/Alaska Native (AIAN) have a large majority of US counties with populations smaller than 1%, and thus do not provide an adequate statistical power for detecting potential effects in our regression analysis. We kindly ask the reviewer to check Section 1.1 in the Appendix for a more elaborate description. We also have stated this as a limitation of our study in the Limitations section.

6) The rationale for selecting specific variables in the PBL model is unclear. COPD and age65+ are reasonable covariates in the context of COVID-19, but other relevant factors, such as additional chronic conditions (e.g., obesity) or sex, are omitted. The authors should provide a more detailed epidemiological rationale for the inclusion of these covariates and the exclusion of others.

We have carried out additional experiments to include nine known comorbidities of COVID-19 based on the CDC [1]. Specifically, we have included in our regression model the following covariates: high blood pressure, cancer, diabetes, obesity, stroke, chronic obstructive pulmonary disease (COPD), Chronic kidney disease (CKD), current asthma, and coronary heart disease (CHD). Our inclusion was also contingent on the availability of county-segregated Health Outcomes Data provided by CDC-PLACES dataset [2], which provides the Age-Adjusted Health Outcomes. We kindly ask the reviewer to check the newly edited “Control variables” section in the main paper and our edited Results section to check all of our new outcomes.

[1] https://www.cdc.gov/covid/hcp/clinical-care/underlying-conditions.html
[2] https://www.cdc.gov/places/index.html

7) Similarly, it is unclear why race is the only covariate considered in the fairness analysis. The manuscript might be more accurately described as addressing racial fairness rather than fairness (broadly). Fairness-related variables, such as gender/sex, poverty, social vulnerability, and age, are not considered.

We focus on two critical dimensions of fairness analysis - **race/ethnicity and urbanization levels** - rather than attempting to cover all possible fairness-related variables. This focused approach is well-justified for several reasons.

First, the COVID-19 pandemic has revealed stark disparities in healthcare outcomes specifically across racial/ethnic groups [1] and the urban-rural spectrum [2], as extensively documented in prior research. Second, there is substantial evidence of systematic data collection and reporting biases affecting these particular groups during the pandemic. For example, as mentioned in the

Introduction, testing has been demonstrably inadequate for certain minority communities and rural areas, leading to significant underreporting of cases.

While other fairness-related variables like gender, poverty, social vulnerability, and age are undoubtedly important, our analysis framework prioritized examining the disparities that have been most prominently documented in COVID-19 outcomes and data collection. However, it's worth noting that our methodological approach is extensible and could be applied to analyze additional fairness-related variables as a future work.

[1]
https://www.cdc.gov/nchs/nvss/vsrr/covid19/health_disparities.htm#CountyRaceHispanicOrigin
[2] Souch, J. M., & Cossman, J. S. (2020). A commentary on rural‐urban disparities in COVID‐19 testing rates per 100,000 and risk factors. The Journal of Rural Health, 37(1), 188.

Changes to the paper: We have reworded social determinants to focus on racial and urbanization level. We have also explicitly mentioned the sensitive attributes we are analyzing in this study in Section: Sensitive Attributes in Material and Methods.

8) It would be useful to include more detailed information in the appendix to assess model fit, such as ANCOVA tables with corresponding statistics. The finding that all regression coefficients were statistically significant is intriguing…

As stated earlier, and for clarity purposes, we have now focused our analysis on the regression approach only. This has provided us with more space to discuss in-depth model selection and diagnostics for the regression. We kindly ask the reviewer to check the "Model Selection and Evaluation" section in the main paper (page 10) as well as to Section 2 in the S1 Appendix.

10) This is related to 8) in that ANCOVA's assumptions aren't tested for (e.g. linearlty, normality etc) in each of the ANCOVAs. ANCOVA is a model and it has its assumptions. If the assumptions aren't met we, readers, cannot conclude whether what we are seeing as results is correct or not. You can consult "ANOVA and ANCOVA: a GLM" approach for example.

As stated in our previous answer, we have now provided an in-depth description of model selection, model diagnostics and assumptions to validate model correctness for the regression approach. We refer the reviewer to the "Model Selection and Evaluation" section in the main paper (page 10) as well as to Section 2 in the S1 Appendix.

9) In a study centered on fairness, it is worth highlighting the exclusion of U.S. territories like Puerto Rico from the evaluation. The manuscript appears to exclude Puerto Rico from both the U.S. map and the county count. This exclusion omits a significant Hispanic population, and the authors should explain the rationale for this decision.

We have added a discussion in Section 1.1 in S1 Appendix, where we justify the exclusion of certain counties from our analysis. In the case of Puerto Rico (FIPS CODE: 72XXX), the CDC

ForecastHub did not provide county-level forecasts for this region, and hence we could not include it in our fairness analysis. Unfortunately, we do not know why the CDC did not provide forecasts for Puerto Rico.

11) Lastly, there is an inconsistency in the number of counties reported in the Supplementary Appendix. Table 2 lists 3,144 counties, while Tables 3 and 4 list 3,142 counties. Please clarify the discrepancy.

As mentioned earlier, we have carried out additional experiments to include known comorbidities of COVID-19 extracted from the CDC-PLACES dataset. After this change in our analysis, and due to the dependency on the availability of data for the various data sources used in this study, we ended up focusing our analysis on 3,067 counties throughout the paper. We have provided additional details in Section 1.1 in the S1 Appendix.
